# A 4-guanidinobutanoic acid-SLC36A1 axis drives a microbiota‒host feedback loop to regulate intestinal homeostasis

**DOI:** 10.1080/19490976.2026.2639216

**Published:** 2026-03-04

**Authors:** Jianming Yang, Yawen Xiao, Jifang Cui, Ruofan Song, Wanxia Ma, Jiangpeng Liu, Chunhui Miao, Xinyu Sun, Xueting Kong, Zhi-Song Zhang, Lu Zhou, Zhi Yao, Quan Wang

**Affiliations:** a Department of Immunology, Tianjin Institute of Immunology, Key Laboratory of Immune Microenvironment and Disease (Ministry of Education), School of Basic Medical Sciences, Tianjin Medical University, State Key Laboratory of Experimental Hematology, Chinese Academy of Medical Sciences and Peking Union Medical College, Tianjin, China; b State Key Laboratory of Medicinal Chemical Biology and College of Pharmacy, Tianjin Key Laboratory of Molecular Drug Research, Nankai University, Tianjin, China; c Institute of Medicinal Biotechnology, Chinese Academy of Medical Sciences and Peking Union Medical College, State Key Laboratory of Experimental Hematology, Beijing, China; d Department of Gastroenterology, Tianjin Union Medical Center, The First Affiliated Hospital of Nankai University, Tianjin, China; e Department of Basic Medical Sciences, Tianjin Medical College, Tianjin, China; f Department of Gastroenterology and Hepatology, Tianjin Medical University General Hospital, Tianjin Medical University, Tianjin, China

**Keywords:** 4-Guanidinobutanoic acid, SLC36A1, intestinal mucosal barrier, *Akkermansia muciniphila*, ulcerative colitis

## Abstract

The role of gut microbiota‒derived metabolites in regulating the intestinal mucosal barrier remains poorly defined. Here, we identified 4-guanidinobutanoic acid (4-GBA), produced by *Bacteroides stercorirosoris*, as a critical regulator of intestinal homeostasis. Using untargeted metabolomics, organoid co-cultures, mouse models, and single-cell RNA sequencing, we demonstrated that 4-GBA enhances intestinal stem cells (ISCs) function and goblet cell differentiation. This promotes *Akkermansia muciniphila* enrichment through mucus-dependent niche expansion, establishing a microbiota‒host feedback loop. Mechanistically, 4-GBA upregulates the proton-coupled amino acid transporter SLC36A1 and activates the Hedgehog signaling pathway to drive epithelial reprogramming. Clinically, SLC36A1 expression inversely correlates with ulcerative colitis (UC) severity in human samples. Furthermore, the SLC36A1 agonist sarcosine enhances barrier homeostasis and attenuates colitis in mice, highlighting the diagnostic and therapeutic potential of this axis in UC. Our findings reveal a novel microbiome–host axis through which a microbial metabolite modulates epithelial function and microbial ecology, offering a potential therapeutic strategy targeting microbiota–epithelial crosstalk for UC management.

## 1. Introduction

The gut microbiota is a central modulator of host homeostasis and disease pathogenesis, particularly in inflammatory bowel disease (IBD).^1^^,^^2^ Although growing evidence indicates that microbiota–derived metabolites serve as critical signaling mediators orchestrating host-microbe crosstalk,^3-8^ the complex interplay between specific microbial metabolites and host physiological systems remains incompletely understood.

The intestinal mucosal barrier-comprising the epithelium, its secretory products, and commensal microbiota-forms a multilayered system essential for homeostasis.^9^ Lgr5⁺ intestinal stem cells (ISCs) orchestrate rapid epithelial renewal (every 3–5 d) and regeneration following injury by differentiating into absorptive enterocytes, mucin-secreting goblet cells, and other specialized lineages.^10^^,^^11^ Goblet cells secrete mucins to form a stratified mucus layer that physically separates the luminal microbiota from the epithelial surface.^12^^,^^13^ In turn, the microbiota reinforces barrier integrity and mucosal immunity through metabolite-mediated signaling and competitive exclusion of pathogens.^14-17^

4-Guanidinobutanoic acid (4-GBA) is an arginine-derived metabolite originating from both host and microbial sources and is detectable in various human biofluids. Previous studies have reported that 4-GBA can suppress gastric lesions and mediate lifespan extension in *C. elegans* under oxidative and disease-induced stress.^18^^,^^19^ Despite these observations, its functional mechanisms, particularly in intestinal physiology, remain poorly characterized. SLC36A1, a proton-coupled amino acid transporter responsible for 4-GBA uptake, is apically expressed in intestinal epithelia under physiological conditions;^20-22^ however, its functional significance in colonic homeostasis is undefined.

In this study, we demonstrated that commensal-derived 4-GBA sustains intestinal barrier homeostasis via SLC36A1-dependent augmentation of ISCs activity and goblet cell differentiation, which in turn amplifies *Akkermansia muciniphila* abundance. This establishes a positive feedback loop wherein a microbial metabolite modulates host cellular functions to reshape the intestinal microbial ecology. Notably, SLC36A1 expression is significantly reduced in patients with IBD. We further establish SLC36A1 as a critical regulator of ISCs function and microbial homeostasis and show that its activation via sarcosine administration attenuates colitis in mice. These findings identify a previously unrecognized microbiome‒host signaling axis and suggest promising therapeutic strategies for colonic inflammatory disorders through the modulation of microbiota‒host interactions.

## 2. Materials and methods

### 2.1. Mice

This study utilized transgenic and knockout mouse models (Lgr5-CreERT2, H11-CAG-LSL-tdTomato, and Muc2^ΔIEC^) and their wild-type counterparts, all on a C57BL/6J genetic background. The mutant mouse models were generated by GemPharmatech Co., Ltd. (Jiangsu, China). Wild-type C57BL/6J mice were acquired from the Academy of Military Medical Sciences (Beijing, China). All animal experiments were approved by the ethical committees of Tianjin Medical University (TMUaMEC2024034) and Tianjin University of Traditional Chinese Medicine (TCM-LAEC2025251M2101). The mice were housed under specific pathogen-free (SPF) conditions at Tianjin Medical University. For the *Citrobacter rodentium* infection experiments, a separate cohort was housed in an Animal Biosafety Level 2 (ABSL-2) laboratory at Tianjin University of Traditional Chinese Medicine to ensure biosecurity. The experiments uniformly employed 6–8-week-old mice unless specifically stated.

### 2.2. Human samples

Fecal samples were obtained from healthy volunteers with approval from the Ethics Committee of Tianjin Beichen Hospital (no. 2023032908). Colonic tissues were collected from patients with ulcerative colitis (UC) at Tianjin Union Medical Center or Tianjin Medical University General Hospital following approval by their respective Ethics Committees (2023-B30 and IRB2020-KY-190). All participants provided informed consent. The UC diagnosis was verified by colonoscopy and histopathology, and key clinical characteristics are provided in Table S1.

### 2.3. Colonic organoid culture

Colonic fragments (3–5 mm) were subjected to extensive ice-cold PBS (Meilun Biotechnology, Dalian, China) washes until clear. Crypt dissociation was performed by incubation in 2 mM EDTA/PBS (Solarbio Technology, Beijing, China) on ice for 30 min, with subsequent mechanical release via vigorous pipetting in PBS/0.1% BSA (White Shark Biotechnology, Hefei, China). The crypt suspension was filtered through a 70 μm strainer (Falcon, Corning, NY, USA), and the enriched fractions were pelleted by centrifugation (Eppendorf, Germany) at 200 × *g* for 3 min at 4 °C.

Following verification and quantification, the crypts were embedded in Matrigel® GFR Basement Membrane Matrix (Corning). Aliquots of 40 μL (~300 crypts/dome) were plated in a 24-well plate and polymerized at 37 °C for 30 min. Each dome was then supplemented with 500 μL of complete organoid medium (Absin Bioscience, Shanghai, China). Cultures were maintained in a humidified incubator (Thermo Fisher Scientific, USA) at 37 °C with 5% CO₂, with a full medium change every 2–3 d.

### 2.4. Collection of bacterial culture supernatants

Bacterial isolates were streaked onto GAM agar plates (Haibo Biotechnology, Qingdao, China) and incubated at 37 °C under anaerobic conditions (anaerobic pouches; Haibo Biotechnology, Qingdao, China) until single colonies formed. An isolated colony was used to inoculate a liquid GAM broth culture, which was grown to the logarithmic phase as determined by OD₆₀₀ monitoring. Log-phase bacteria (∼6 × 10⁸ cells) were collected by centrifugation, washed twice with PBS, and resuspended in organoid medium for overnight metabolite secretion at 37 °C. The conditioned medium was centrifuged, and the supernatant was sequentially passed through a 0.22 μm filter (White Shark Biotechnology, Hefei, China) and then subjected to ultrafiltration using a 3 kDa molecular weight cut-off device (Millipore, Sigma, St. Louis, MO, USA) at 7500 × *g* for 10 min to isolate the low-molecular-weight fraction. The final filtrates were stored at −80 °C for subsequent analysis.

### 2.5. Treatment of colonic organoids with bacterial supernatant

After 24 h of pre-treatment, mature colon organoids were harvested, re-embedded in Matrigel, and seeded into 24-well plates (100–200 organoids/well). Cultures were stabilized by adding 450 μL of organoid medium (Keluojie Biotechnology, Guangzhou, China) followed by 24 h of incubation (37 °C/5% CO₂). Upon confirmation of structural integrity, 150 μL sterile < 3 kDa bacterial filtrate was added per well. During the 6-d co-culture, half-medium changes were performed every 48 h with concurrent addition of 150 μL fresh < 3 kDa filtrate. Organoids were then harvested for RT-qPCR and immunofluorescence.

### 2.6. DSS-induced colitis

Mice received 2% (w/v) dextran sulfate sodium (DSS; MP Biomedicals, USA) in drinking water for 7 d to induce acute colitis. Disease activity was assessed daily using a composite disease activity index (DAI) based on weight loss, stool consistency, and fecal blood. On day 7, the mice were sacrificed, and the distal colon was harvested and segmented for four parallel downstream applications: (1) fixation in 4% PFA and paraffin embedding for histology; (2) embedding in OCT compound (Scigen Scientific, USA) and snap-freezing; (3) direct snap-freezing for RNA and cytokine analysis; and (4) isolation of lamina propria immune cells for flow cytometry.

### 2.7. Oral administration of 4-GBA, forskolin, and sarcosine in mice

4-Guanidinobutanoic acid (4-GBA; Sigma-Aldrich; 150 mg/kg) and forskolin (Selleck, Houston, TX, USA; 100 mg/kg) were freshly dissolved in sterile PBS and administered via daily oral gavage for seven consecutive days. Separately, sarcosine (500 mg/kg; MedChemExpress, Monmouth Junction, NJ, USA), an SLC36A1 inducer, was administered as a single gavage dose in normal saline.

### 2.8. *C. rodentium* infection *in vivo*

Mice were infected with a nalidixic acid-resistant *C. rodentium* strain following a 12-h fast. The inoculum (1 × 10⁹ CFU in 200 μL PBS) was prepared from an overnight LB broth culture that had been washed and resuspended. Post-infection, the mice were treated daily with 4-GBA (150 mg/kg via oral gavage) for 14 d. Upon termination, bacterial loads in colon homogenates and feces were determined by plating serial dilutions on nalidixic acid (10 μg/mL)-containing LB agar. Parallel colon segments were processed for H&E staining and subsequent histopathological evaluation of inflammation and tissue damage.

### 2.9. H&E and AB–PAS staining

Distal colon segments were fixed in 4% paraformaldehyde for 24 h, processed through a standard automated tissue processor, and embedded in paraffin. Subsequently, 5 µm-thick sections were prepared for staining. For the general histopathological assessment, the sections were stained with hematoxylin and eosin (H&E) and evaluated using a comprehensive scoring system that encompassed seven parameters: inflammation depth, crypt damage severity, immune cell infiltration, submucosal edema, goblet cell depletion, epithelial hyperplasia, and crypt abscess formation. For specific quantification of goblet cells, parallel sections were stained with Alcian Blue–Periodic Acid-Schiff (AB–PAS) using a commercial kit (Solarbio) per the manufacturer's instructions. The number of AB–PAS-positive goblet cells was determined by counting the number of cells within the crypts and organoids across four randomly selected high-power fields per section.

### 2.10. Immunohistochemistry

Paraffin-embedded tissue sections (5 μm) were deparaffinized in xylene, rehydrated through a graded ethanol series, and subjected to heat-induced antigen retrieval in citrate buffer (pH 6.0; ZSGB-BIO, Beijing, China). The endogenous peroxidase activity was subsequently quenched with 3% hydrogen peroxide, and non-specific binding sites were blocked with normal serum. The sections were then incubated overnight at 4 °C with the following primary antibodies: MUC2 (1:600, Proteintech, Wuhan, China), SLC36A1 (1:2000, Proteintech), Gli1 (1:2000, Proteintech), cleaved Caspase-3 (1:2000, Cell Signaling Technology, Danvers, MA, USA), occludin (1:200, Abcam, Cambridge, UK), Ki67 (1:50, Abcam), CHGA (1:1000, Abcam), and DCLK1 (1:1000, Abcam). The following day, the sections were incubated with appropriate HRP-conjugated secondary antibodies, and immunoreactivity was visualized using 3,3′-diaminobenzidine (DAB) as the chromogen. Finally, the sections were counterstained with hematoxylin, dehydrated, cleared, and coverslipped. Stained images were captured using an Olympus BX46 microscope, and the staining intensity was quantified with Image Pro Plus software (Media Cybernetics, Rockville, MD, USA).

### 2.11. Immunofluorescence

Immunofluorescence staining was performed on organoids, murine colonic tissues, and human colonic tissues with the following distinct sample preparation and staining procedures. For immunofluorescence staining of the organoids, the organoids were fixed in 4% PFA for 30 min at room temperature, embedded in 3% low-melting-point agarose, and processed into paraffin blocks using an automated tissue processor (Leica Biosystems). Sections (8 µm) were deparaffinized and rehydrated prior to heat-induced antigen retrieval in citrate buffer (pH 6.0; ZSGB-BIO) using an autoclave (121 °C, 15 min). For immunofluorescence staining of murine colonic tissue, distal colon tissues were fixed in 4% PFA for 24 h at 4 °C, cryoprotected in 30% sucrose, embedded in OCT compound (Scigen Scientific), and cryosectioned at 8 µm. The sections were post-fixed in 4% PFA for 10 min before antigen retrieval in citrate buffer at 95 °C for 20 min. For immunofluorescence staining of human colonic tissue, the tissues were rapidly frozen in OCT compound and cryosectioned at 5 µm. The sections were air-dried, fixed in pre-cooled acetone at 4 °C for 10 min, and endogenous peroxidase activity was quenched with pre-cooled 3% H₂O₂ in methanol.

Following sample-specific preparation, all the sections underwent a common staining protocol: permeabilization with Triton X-100 (0.25%–0.3% in PBS) for 15 min, blocking with 5% BSA in PBS for 1 h at room temperature, and incubation with primary antibodies overnight at 4 °C. The primary antibodies used were: anti-MUC2 (1:600; Proteintech) and anti-Ki67 (1:100; Abcam) for organoids; anti-TdTomato (1:400; Biorbyt, San Francisco, CA, USA) and anti-Ki67 (1:100; Abcam) for murine tissue; and anti-Gli1 (1:500; Abcam) and anti-SLC36A1 (1:1000; Abcam) for human tissue. The sections were subsequently incubated with appropriate fluorophore-conjugated secondary antibodies (Alexa Fluor 488/594; 1:500) for 1 h at room temperature and counterstained with DAPI. All images were acquired using a confocal microscope (Leica TCS-SP8, Leica Microsystems, Germany).

### 2.12. Lineage tracing of Lgr5^+^ intestinal stem cells

Lineage tracing of Lgr5⁺ intestinal stem cells was performed using Lgr5-CreERT2; H11-CAG-LSL-tdTomato transgenic mice. Cre activity was induced by administering 4-hydroxytamoxifen (4-OHT; 20 mg/kg, i.p.; Sigma-Aldrich) daily for three days, with the compound dissolved in corn oil (20 mg/mL).

### 2.13. Induction of tdTomato expression in colonic organoids *in vitro*

Colonic organoids derived from Lgr5-CreERT2; H11-CAG-LSL-tdTomato mice were utilized for *in vitro* lineage tracing of Lgr5⁺ intestinal stem cells. The organoids were treated with 2 μM 4-hydroxytamoxifen (from a 10 mM DMSO stock) in IntestiCult™ medium for 72 h to induce Cre recombination.

### 2.14. Pharmacological inhibition of signaling pathways in colonic organoids

To delineate the role of specific signaling pathways in regulating the murine colonic epithelium, we treated the colonic organoids with selective pharmacological inhibitors. The organoids were exposed for 48 h to the following compounds, each dissolved in DMSO and diluted in culture medium: SQ22536 (100 μM; Selleck), an inhibitor of the cAMP pathway; cyclopamine (1 μM; Selleck), a Hedgehog pathway inhibitor; AS1842856 (1 μM; Selleck), a Foxo inhibitor; and IWP-2 (20 μM; Selleck), an inhibitor of Wnt signaling.

### 2.15. Total RNA isolation and quantitative real-time PCR

Total RNA was isolated from colonic organoids and tissues using a commercial extraction kit (Solabao Technology) following the manufacturer's instructions. Subsequently, 1 µg of the purified RNA was reverse-transcribed into cDNA using the RevertAid First Strand cDNA Synthesis Kit (Thermo Fisher Scientific). Quantitative real-time PCR (qRT-PCR) was then performed in triplicate on a QuantStudio 1 system (Thermo Fisher Scientific) with ChamQ Universal SYBR qPCR Master Mix (Vazyme, Nanjing, China). The relative expression of the target genes was normalized to that of β-actin as an internal control and calculated using the 2^−ΔΔCt^ method. All primer sequences are listed in Supplementary Table S2.

### 2.16. Antibiotic treatment

To deplete the gut microbiota, the mice were administered a broad-spectrum antibiotic cocktail in their drinking water *ad libitum* for seven consecutive days. The cocktail consisted of neomycin (1 g/L), ampicillin (1 g/L), metronidazole (1 g/L), and vancomycin (0.5 g/L). The antibiotic solution was freshly prepared and replaced every 3‒4 d to ensure stability and efficacy.

### 2.17. Fecal microbiota transplantation (FMT)

To investigate the role of the gut microbiota, fecal microbiota transplantation (FMT) was performed using donor feces from WT mice or 4-GBA-treated mice. Fresh fecal pellets (200 mg) were homogenized in sterile anaerobic PBS daily. The homogenate was centrifuged at 800 × *g* for 3 min, and the resulting supernatant was filtered through a 70-μm strainer to prepare the fecal suspension. Recipient WT mice were first subjected to a 7-d broad-spectrum antibiotic regimen to deplete their endogenous gut microbiota. Following this depletion, the mice received a daily intragastric gavage of 200 μL of the prepared fecal suspension for seven days. Subsequently, acute colitis was induced in these recipients by administering 2% DSS in their drinking water for an additional seven days.

### 2.18. Non-targeted metabolomics analysis

The bacterial culture metabolites in the <3 kDa fraction were lyophilized and extracted with cold methanol-water containing an internal standard (L-2-chlorophenylalanine). The clarified extract was analyzed by untargeted metabolomics (Majorbio Bio-Pharm, Shanghai, China), utilizing UHPLC-Q Exactive HF-X MS with dual ESI polarity mode and QC monitoring. Data processing (Progenesis QI) included peak alignment, feature extraction, and data filtering. Metabolite annotation was performed against the HMDB, METLIN, and Majorbio databases, followed by statistical and pathway analysis on the I-Sanger Cloud platform. The non-targeted metabolomics raw data are available through the Metabolomics Workbench project ID PR002560.

### 2.19. Targeted metabolomics analysis

Fecal samples from mice and human subjects were weighed (10 mg) and processed for targeted metabolomics analysis at Majorbio Bio-Pharm (Shanghai, China). The samples were homogenized with pre-cooled 50% methanol-water containing isotope-labeled internal standards using a frozen tissue grinder. After centrifugation, the supernatant was derivatized with 3-nitrophenylhydrazine (3NPH) and N-(3-dimethylaminopropyl)-N′-ethylcarbodiimide (EDC) at 40 °C for 60 min. The derivatized samples were analyzed by targeted LC‒MS/MS (SCIEX QTRAP 6500+) using a Waters HSS T3 column (2.1 × 150 mm, 1.8 μm) with a methanol/water gradient (both containing 0.03% formic acid) and negative ion ESI mode. The quantification of 4-GBA was performed by comparing the peak area ratio (analyte/internal standard) against a multi-point calibration curve, with the QC samples confirming system stability.

### 2.20. Single-cell RNA sequencing analysis

After a 7-d antibiotic-mediated microbiota depletion, the mice were treated with either 4-GBA or sterile water (control) for 7 d. Viable single-cell suspensions (>90%) from colonic crypts were prepared and used for scRNA-seq library generation (10 × Genomics Chromium). Libraries were quality-checked and sequenced on an Illumina NovaSeq platform. Data processing (Cell Ranger) included alignment, filtering, and matrix generation. The bioinformatics analyses encompassed clustering (Louvain), visualization (UMAP), cell type annotation using marker genes, and KEGG enrichment analysis of differentially expressed genes. All sequencing data are available in the NCBI BioProject databases under the accession number PRJNA1298199.

### 2.21. 16S rRNA gene sequencing

Fecal genomic DNA was extracted from freshly collected pellets using the Omega Bio-tek Stool DNA Kit (D4015-02, Omega Bio-tek, Norcross, GA, USA) and quality-checked by NanoDrop 2000 spectrophotometry (concentration/purity) and agarose gel electrophoresis (integrity). The V3–V4 regions of the 16S rRNA gene were amplified with the primers 515F/806R, and the libraries were sequenced on an Illumina MiSeq platform (Illumina, San Diego, CA, USA) after quantification and pooling. All downstream bioinformatics (OTU clustering, diversity analysis, LEfSe) were conducted on the Majorbio Cloud Platform (https://cloud.majorbio.com/). Raw sequencing reads were deposited in the NCBI BioProject databases under the accession numbers PRJNA1298487 and PRJNA1298490.

### 2.22. Lentiviral-mediated shRNA knockdown and SLC36A1 overexpression in colonic organoids

Lentiviral vectors for SLC36A1 knockdown and overexpression were constructed as follows. For knockdown, shRNAs targeting murine SLC36A1 were designed using the siRNA Target Finder (Ambion) and cloned into the pLKO.1 backbone. For overexpression, the murine SLC36A1 coding sequence was amplified by PCR and cloned into a pITA vector to generate a C-terminal 3 × FLAG-tagged fusion construct.

Lentiviral particles were produced by co-transfecting HEK293T cells with the respective transfer plasmid (shRNA or SLC36A1) along with psPAX2 packaging and pMD2.G envelope plasmids. Viral supernatants were collected 48–72 h post-transfection, filtered through 0.45 μm filters, and concentrated by polyethylene glycol (PEG) precipitation.

To generate stable lines, colonic organoids were dissociated into single cells and transduced with the concentrated lentivirus in the presence of polybrene (6 μg/mL). After removal of the virus, the cells were re-embedded in Matrigel to allow organoid reformation. Transduced organoids were selected under continuous puromycin pressure (2 μg/mL) to establish stable SLC36A1 knockdown or overexpression models.

### 2.23. Human and murine stool-derived *ex vivo* communities

Fecal samples (10 mg) from human donors and WT mice were suspended in anaerobic PBS, homogenized, and centrifuged at 550 × *g* for 5 min to pellet large particles. The supernatant was then harvested, diluted ten-fold, and inoculated (1% v/v) into autoclaved, pre-conditioned Gifu anaerobic medium (GAM; Hopebio) supplemented with 0.01% (w/v) L-cysteine (Sigma-Aldrich) in anaerobic serum bottles. To evaluate the impact of 4-GBA on *A. muciniphila*, these cultures were supplemented with 0.8 mM 4-GBA or vehicle control and incubated anaerobically for 48 h. Post-incubation, *A. muciniphila* abundance was determined by qPCR (targeting the *dnaA* gene), and 16S rRNA sequencing of extracted bacterial DNA.

### 2.24. *A. muciniphila* depletion

To investigate the role of *A. muciniphila*, the mice were administered benzydamine hydrochloride (50 mg/mL in sterile PBS) via oral gavage (200 μL per dose) three times per week for two weeks to deplete this specific bacterium. The efficacy of depletion was confirmed by quantifying *A. muciniphila* abundance in fecal samples using qPCR targeting the species-specific *dnaA* gene. Following successful depletion, acute colitis was induced in these mice by administering 2% (w/v) DSS in their drinking water for 7 d. Disease progression was monitored daily through the disease activity index (DAI).

### 2.25. Isolation of mesenteric lymph nodes (MLN) and colonic lamina propria cells

Mesenteric lymph nodes (MLNs) were aseptically collected and mechanically dissociated through a 70-μm strainer in ice-cold RPMI-1640 medium supplemented with 10% FBS. For lamina propria lymphocytes, the colons were opened, cleaned, and minced. Tissues underwent sequential treatment with a DTT/EDTA pre-digestion buffer (2 mM DTT, 5 mM EDTA) to remove the epithelium, followed by enzymatic digestion (0.5 mg/mL collagenase, 0.05 mg/mL hyaluronic acid, 100 ng/mL DNase I in RPMI-1640; 37 °C, 150 rpm, 30 min). The liberated cells were filtered through a 70-μm strainer, and lamina propria mononuclear cells were subsequently isolated by density gradient centrifugation using 40% Percoll (GE Healthcare, Chicago, IL, USA) at 670 × *g* for 30 min at 4 °C.

### 2.26. Flow cytometry

For cell surface phenotyping, cells were incubated 30 min with the following fluorophore-conjugated antibodies: CD45–APC (1:100, Biolegend, San Diego, CA, USA), CD11b–PE–Cy7 (1:100, Invitrogen, Waltham, MA, USA), F4/80–FITC (1:100, eBioscience, San Diego, CA, USA), MHC II–PE (1:100, Biolegend), CD11c–APC (1:100, Biolegend), CD45–PerCP/Cy5.5 (1:100, Biolegend), and Ly6G APC (1:100, Biolegend).

For intracellular cytokines, cells were stimulated for 5 h at 37 °C with a Brefeldin A-containing activation cocktail (Biolegend), then stained with Zombie NIR™ Fixable Viability Dye (1:1000, Biolegend) for 30 min, followed by surface staining with CD3-FITC (1:100, Biolegend) and CD4-PE-Cy7 (1:100, Biolegend). The cells were then fixed and permeabilized using the Intracellular Staining Permeabilization Wash Buffer Set (BioLegend) according to the manufacturer's instructions and stained with IL-17A-APC (1:100, Biolegend), IFN-γ-PerCP/Cy5.5 (1:100, Invitrogen), and IL-4-PE (1:100, Biolegend).

For Treg analysis, the cells were stained for the surface markers CD3-FITC (1:100, BioLegend), CD4-PE-Cy7 (1:100, Biolegend), and CD25-PE (1:100, Biolegend), followed by fixation and permeabilization using the Foxp3/Transcription Factor Staining Buffer Set (eBioscience, Waltham, MA, USA). Intracellular staining was performed using Foxp3-APC (1:100, Biolegend). Data were acquired on a FACS Canto II (BD Biosciences) and analyzed with FlowJo v10.9 software (FlowJo LLC, Ashland, OR, USA).

### 2.27. GEO datasets analysis

To analyze *SLC36A1* and *Ptch1* gene expression in the colon biopsies of patients with UC, microarray data from the Gene Expression Omnibus (GSE66407 and GSE47908) were processed using the Sanger Box software.

### 2.28. ScRNA-seq analysis

The SLC36A1 expression in colonic epithelial cells was analyzed by Iotabiome Biotechnology Co., Ltd., in the colon biopsies of patients with UC using Gene Expression Omnibus (GSE231993 and GSE214695) and single-cell RNA sequencing (scRNA-seq) data on the Broad Institute Single Cell portal (https://singlecell.broadinstitute.org/single_cell/study/SCP259/intra-and-inter-cellular-rewiring-of-the-human-colon-during-ulcerative-colitis).

### 2.29. Quantification and statistical analysis

Data are expressed as mean ± SD. The statistical significance was assessed using unpaired Student's *t*-tests, paired Student's *t*-tests, one-way ANOVA, or two-way ANOVA. Correlation analyses were conducted using Pearson's correlation. Statistical analyses were performed using GraphPad Prism (version 8.0; GraphPad Software, San Diego, CA, USA).

## 3. Results

### 3.1. Gut commensal bacteria-derived 4-GBA augments goblet cells in colonic organoids

**Figure 1.**
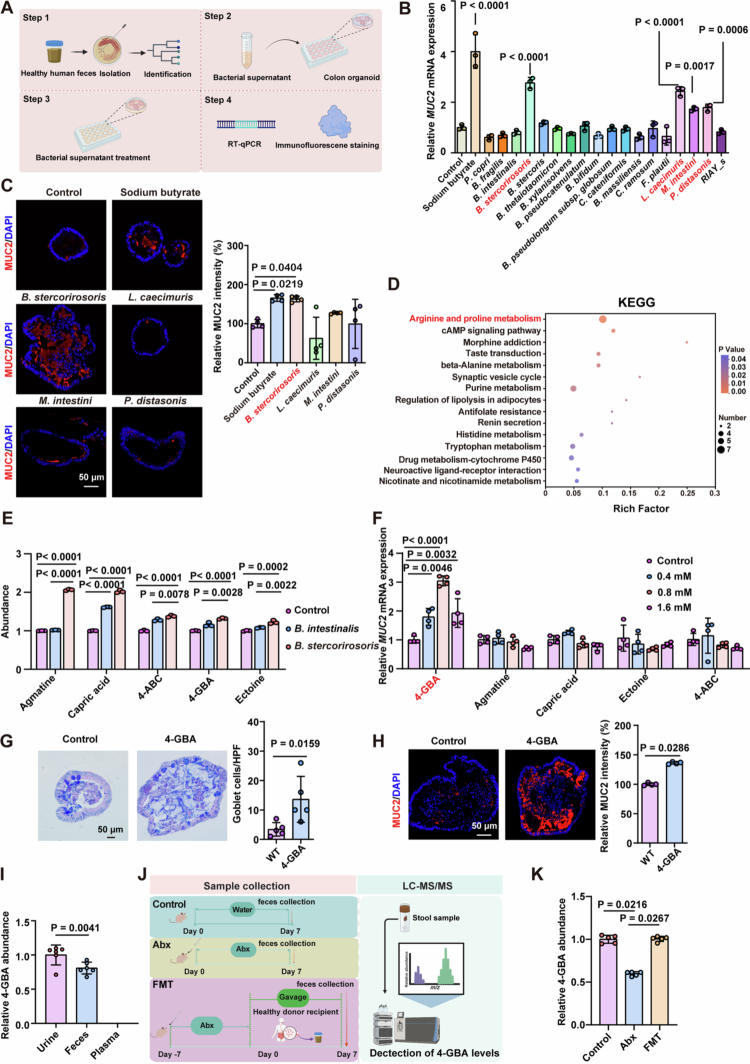
Commensal-derived 4-GBA expands goblet cells in colonic organoids. (A) Schematic representation of colonic organoid treated with fractionated (<3 kDa) metabolites from human gut commensal supernatants. Commensal bacterial strains were isolated from the fecal samples of healthy volunteers and taxonomically identified. Colonic organoids were exposed to <3 kDa fractions of culture supernatants derived from these strains. MUC2 expression was analyzed by qRT-PCR and immunofluorescence staining. Sodium butyrate was used as a positive control. (B and C) Colonic organoids isolated from WT mice were treated with commensal bacterial culture supernatants (25% v/v) for six consecutive days. (B) qRT-PCR analysis of *MUC2* mRNA levels in colonic organoids. (C) Representative images and quantification of immunofluorescence staining against MUC2 in the indicated colonic organoid. (D and E) Untargeted metabolomics analysis of *B. stercorirosoris* supernatant using blank organoid medium and supernatant from the negative control strain *B. intestinalis* as controls. (D) KEGG enrichment analysis of 291 overlapped metabolites. (E) LC‒MS quantification of 4-GBA in the indicated supernatants. (F) qRT-PCR analysis of *MUC2* mRNA levels in colonic organoids following a 4-d treatment with various metabolites at the indicated concentrations. (G and H) Colonic organoids isolated from WT mice were exposed to water (control) and 4-GBA (0.8 mM) for 4 d. (G) Alcian Blue–Periodic Acid Schiff (AB–PAS) staining in colonic organoid. (H) Representative images and quantification of immunofluorescence staining against MUC2 in colonic organoids. (I) LC‒MS quantification of 4-GBA in urine, feces, and plasma from 6 healthy volunteers. (J and K) The mice were assigned to the control (drinking water), Abx (antibiotics in the drinking water), and FMT (transplantation with healthy volunteers fecal microbiota after antibiotic treatment). Fecal samples were collected for 4-GBA quantification. (J) Schematic representation of the experimental workflow. (K) LC‒MS quantification of 4-GBA in fecal samples. The data are the mean ± SD. Scale bar: 50 μm. *n* = 4 (B, C, F, and H), *n* = 3 (E), *n* = 5 (G and K), *n* = 6 (I). One-way ANOVA (B, C, E, F, and K) or unpaired Student's *t*-test (G–I).

To investigate the role of gut microbial metabolites in mucosal barrier regulation, we established a murine colonic organoid co-culture system using <3 kDa-filtered supernatants from human gut commensal bacteria and evaluated their effects on the expression of MUC2 (the key component of the colonic mucus secreted by goblet cells) (Figure 1A). From the fecal samples of healthy volunteers, we isolated 297 bacterial strains representing 36 species based on 16S rRNA gene sequence analysis. Among these, 18 strains exhibited no significant growth inhibition on organoids (Figure S1A). qRT-PCR screening identified four strains that significantly upregulated *MUC2* mRNA levels (Figure 1B). Immunofluorescence analysis revealed that the *Bacteroides stercorirosoris* supernatant induced the most more pronounced MUC2 protein expression compared to the other three positive strains (Figure 1C).

To identify the specific metabolite responsible for MUC2 upregulation, we performed untargeted metabolomics analysis of *B. stercorirosoris* supernatant using two controls: blank organoid medium and supernatant from the negative control strain *B. intestinalis*. Principal component analysis (PCA) revealed distinct metabolic profiles among the three groups (Figure S1B). Comparative analysis identified 645 significantly upregulated metabolites in *B. stercorirosoris* supernatant versus blank medium, and 342 unique metabolites compared to *B. intestinalis* supernatant (Figure S1C and D). A core set of 291 overlapping metabolites (Figure S1E) was further analyzed to elucidate *B. stercorirosoris*-specific metabolic signatures. KEGG pathway enrichment analysis revealed “arginine and proline metabolism” as the most significantly enriched pathway (Figure 1D), highlighting its pivotal role in defining the metabolic characteristics of this bacterial strain. Within this pathway, five key metabolites–agmatine, 4-acetamidobutanoic acid (4-ABC), capric acid, 4-guanidinobutanoic acid (4-GBA), and ectoine-exhibited significantly higher abundance in *B. stercorirosoris* supernatant (Figure 1E). Functional validation demonstrated that treatment with 4-GBA at a concentration of 0.8 mM significantly enhanced both *MUC2* expression and goblet cell numbers in colonic organoids, whereas the other tested metabolites did not (Figure 1F–H).

To assess 4-GBA's physiological relevance, we analyzed its distribution in clinical samples from six healthy volunteers. This metabolite was detected exclusively in the urine and feces, with no detectable presence in the plasma (Figure 1I). To establish microbial origin, fecal 4-GBA levels in feces were compared across three experimental groups: antibiotic-treated mice, control normal mice, and human fecal microbiota transplantation (FMT)-treated mice (Figure 1J). It is demonstrated that antibiotic treatment significantly reduced fecal 4-GBA levels compared to controls, while FMT treatment effectively restored these levels (Figure 1K), providing direct evidence that the gut microbiota are the important source of this metabolite.

### 3.2. 4-GBA promotes intestinal homeostasis through enhanced stem cell function and barrier protection

**Figure 2.**
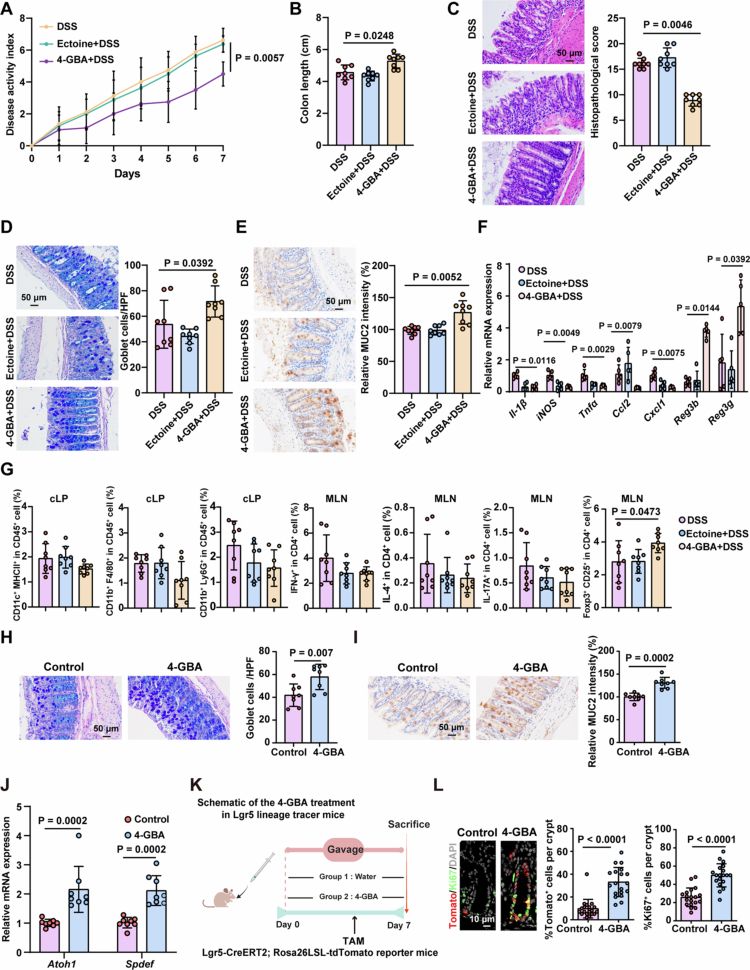
4-GBA attenuates DSS-induced colitis. (A–G) Mice received 2% DSS in drinking water for 7 d with co-administration of either 4-GBA (150 mg/kg) or Ectoine (150 mg/kg). Analysis of disease activity index scores (A) and colon length (B) of the indicated mice. (C) Representative H&E staining analysis and quantitation of histopathological changes in colonic tissue. (D) AB–PAS staining and quantitation of colonic tissue. (E) Representative immunohistochemical analysis and quantitation of MUC2 in colonic tissue. (F) mRNA expression levels of proinflammatory cytokines and antimicrobial peptides in the colonic tissue. (G) Flow cytometry analysis of the indicated cells in mesenteric lymph nodes (MLN) and the colonic lamina propria (cLP) from the indicated mice. (H–J) WT mice received daily oral gavage of 4-GBA (150 mg/kg) for seven consecutive days. AB–PAS staining (H) and immunohistochemical analysis of MUC2 (I) in colonic tissue. (J) mRNA expression levels of *Atoh1* and *Spdef* in the colonic tissue. (K and L) Lgr5-Tomato reporter mice received oral 4-GBA (150 mg/kg/d) or vehicle control for seven consecutive days. Tamoxifen (20 mg/kg/d, i.p.) was administered on days 5–7 to label Lgr5-Tomato^+^ cells. (K) Schematic of the workflow. (L) Representative images and quantification of the percentages of Tomato^+^ crypts and Ki-67^+^ cells among total crypts in mouse colon tissue. *n* > 15 crypts per measurement, *n* > 4 measurements per mouse, and *n* = 5 mice per group. The data are the mean ± SD. Scale bar: 10 μm or 50 μm. *n* = 8 (A–E, G–J), *n* = 5 (F and L). Two-way ANOVA (A), one-way ANOVA (B‒G) or unpaired Student's *t*-test (H–J, L).

To evaluate the effect of 4-GBA in improving the mucosal barrier, we employed two murine models: DSS-induced colitis and *C. rodentium*-induced colitis. In DSS-treated mice receiving 4-GBA or Ectoine (negative control) administration, the 4-GBA group exhibited significantly reduced disease activity index (DAI) scores compared to controls (Figure 2A). Longer colons and attenuated histopathological severity in 4-GBA-treated mice were observed (Figure 2B and C). Given the characteristic goblet cell depletion in colitis, we quantified the goblet cell counts and MUC2 expression in colon tissues. 4-GBA treatment resulted in significantly increased goblet cell numbers and elevated MUC2 levels relative to those in WT controls (Figure 2D and E). Quantitative assessment of inflammatory markers showed reduced expression of proinflammatory cytokines (*Il-1β*, *iNOS*, *Tnfα*, *Ccl2*, and *Cxcl1*) and enhanced expression of antimicrobial peptides (*Reg3g* and *Reg3b*) in 4-GBA-exposed colon tissues (Figure 2F). Flow cytometric analysis of colonic immune infiltrates revealed no major changes in macrophages, dendritic cells, neutrophils, or Th1/Th2/Th17 cell populations (Figures 2G and S1F). However, a significant increase in regulatory T cell (Treg) frequency was observed in the 4-GBA group (Figures 2G and S1F). This shift in the Treg compartment is consistent with an immune environment shaped secondarily by restored mucosal homeostasis. Collectively, these results indicate that 4-GBA attenuates DSS-induced colitis. Consistently, 4-GBA ameliorates *C. rodentium*-induced colitis phenotype, as evidenced by reduced intestinal damage (Figure S1G–H) and lower *C. rodentium* colony-forming units (CFUs) in feces and colon tissue (Figure S1I–J).

To elucidate the mechanisms underlying these protective effects, we evaluated the baseline impact of 4-GBA on epithelial homeostasis. Histopathological examination of colon, liver, kidney, and lung tissues showed no significant differences between the 4-GBA-treated and control mice (Figure S1K), and colonic length remained unchanged (Figure S1L). Furthermore, to directly address the physiological relevance of our dosing strategy, we quantified the fecal 4-GBA concentrations following oral administration. Fecal 4-GBA levels were significantly elevated in treated mice Compared to vehicle-controls (Figure S1M). Notably, the concentrations achieved exceeded the physiological range detected in fecal samples from healthy human donors (Figure S1M), supporting the potential for a therapeutic window in ulcerative colitis. Moreover, 4-GBA exposure significantly increased goblet cell counts and MUC2 protein levels compared to controls (Figure 2H and I). This goblet-biased differentiation was selective, as no change was observed in CHGA-positive enteroendocrine cells (Figure S1N), and tuft cell marker expression was modestly decreased (Figure S1O). At the transcriptional level, 4-GBA upregulated *Atoh1*, a master regulator of secretory fate, and *Spdef*, a goblet-cell-specific driver, which is consistent with lineage commitment toward mucin-producing cells (Figure 2J). 4-GBA administration increased Ki67^+^ proliferating cell counts (Figure S1P) without altering cleaved caspase-3^+^ apoptotic cell counts (Figure S1Q) and significantly elevated OCCLUDIN expression in the colon (Figure S1R). Using Lgr5-CreERT2; Rosa26LSL-tdTomato reporter mice (which permanently label ISCs and their progeny with tdTomato following tamoxifen administration), we demonstrated that 4-GBA treatment increased both the proportion of tdTomato^+^ ISCs and the number of Ki67^+^ proliferating cells per intestinal crypt (Figure 2K and L). These data suggest that 4-GBA enhances intestinal epithelial regeneration through ISCs activation.

Collectively, our findings demonstrate that 4-GBA augments intestinal stem cell proliferation, increases goblet cell density, and strengthens mucosal barrier integrity to promote epithelial homeostasis.

### 3.3. 4-GBA alleviates colitis in a gut microbiota-dependent manner

**Figure 3.**
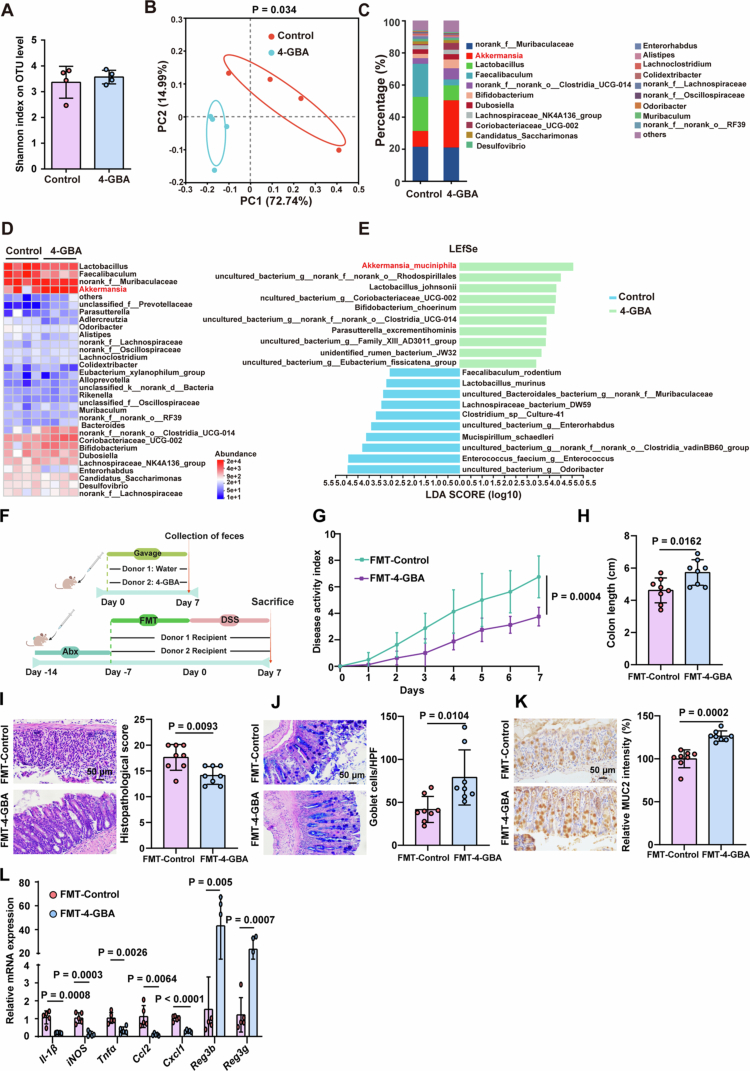
4-GBA ameliorates colitis through microbiota-dependent mechanisms. (A–E) Stool samples from control and 4-GBA-exposed mice were collected and analyzed by 16S rRNA gene sequencing. (A) Shannon index of the gut microbiota. (B) Principal component analysis (PCA) of the fecal microbiota. (C) Percent of bacterial community abundance at the genus level. (D) Heatmap showing the top 30 bacteria at the genus level. (E) LEfSe analysis of distinctive microbiota composition at the species level. (F–I) Antibiotic pretreated mice received fecal microbiota transplantation (FMT) from water control and 4-GBA exposed mice for 7 d, followed by 2% DSS challenge for 7 d. (F) Schematic of the FMT experimental design. (G and H) Analysis of disease activity index scores (G) and colon length (H) of the indicated mice. (I) Representative H&E staining analysis and quantitation of histopathological changes in colonic tissue. (J) AB–PAS staining and quantitation of colonic tissue. (K) Representative immunohistochemical analysis and quantitation of MUC2 in colonic tissue. (L) mRNA expression levels of proinflammatory cytokines and antimicrobial peptides in the colonic tissue. The data are the mean ± SD. Scale bar: 50 μm. *n* = 4 (A–E), *n* = 8 (G–K), and *n* = 5 (L). Two-way ANOVA (G) or unpaired Student's *t*-test (A, H–L).

To determine whether the effects of 4-GBA involve microbiome modulation, we performed 16S rRNA gene sequencing under baseline conditions. While Shannon diversity indices remained comparable between 4-GBA-treated and control mice (Figure 3A), principal coordinates analysis (PCoA) revealed distinct microbial composition profiles (Figure 3B). Taxonomic analysis identified significant enrichment of the *Akkermansia* genus in 4-GBA-treated mice (Figure 3C and D). LEfSe analysis further confirmed *A. muciniphila* as the most differentially abundant species in the 4-GBA group (Figure 3E). During colitis, 4-GBA-treated mice similarly exhibited distinct microbial compositions and elevated *Akkermansia* levels versus controls (Figure S2A–D).

To establish microbiome dependency, mice received broad-spectrum antibiotics (Abx) to deplete the gut microbiota prior to DSS-induced colitis and 4-GBA administration (Figure S2E). This treatment completely abolished 4-GBA's protective effects, with no differences observed in the disease activity index, colon length, histopathological scores, goblet cell counts, or MUC2 expression between the groups (Figure S2F–J). In addition, the fecal microbiota from 4-GBA-treated mice was transplanted into wild-type recipient mice (Figure 3F). Recipients of the 4-GBA-modified microbiota exhibited significantly reduced colitis severity (Figure 3G–L). The combined evidence from these experiments supports a microbiome-dependent mechanism for 4-GBA in colitis mitigation.

### 3.4. *A. muciniphila* mediates 4-GBA's therapeutic effects in colitis

**Figure 4.**
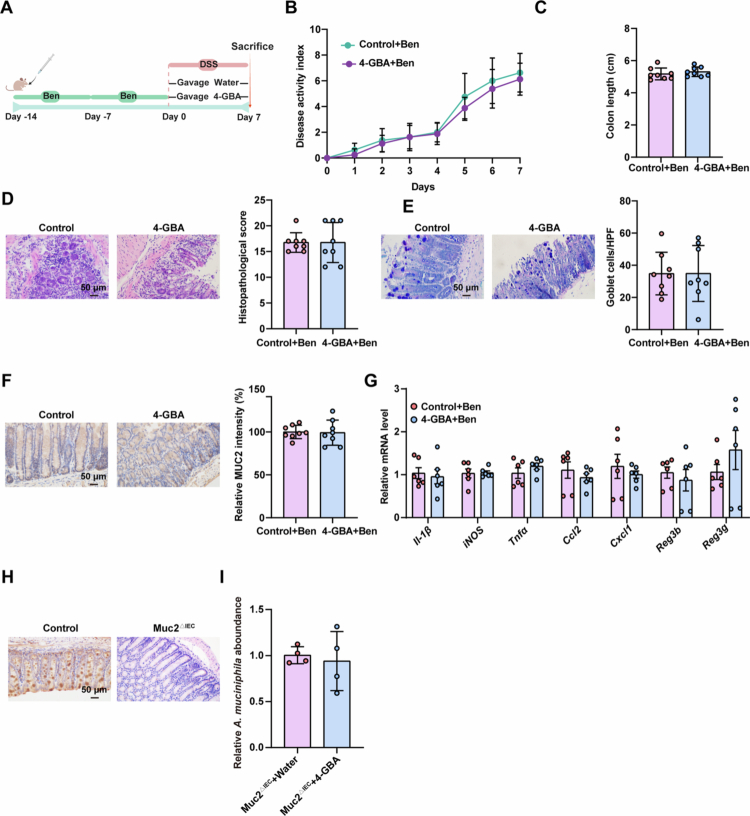
The protective effect of 4-GBA on colitis is dependent on *A. muciniphila.* (A–G) Mice were orally gavaged with benzydamine hydrochloride (50 mg/kg) three times per week for two consecutive weeks, followed by water (control) and 4-GBA (150 mg/kg) exposure plus 2% DSS challenge for 7 d. (A) Schematic of *A. muciniphila* depletion experimental design. (B and C) Analysis of disease activity index scores (B) and colon length (C) of the indicated mice. (D) Representative H&E staining analysis and quantitation of histopathological changes in colonic tissue. (E) AB–PAS staining and quantitation of colonic tissue. (F) Representative immunohistochemical analysis and quantitation of MUC2 in colonic tissue. (G) mRNA expression levels of proinflammatory cytokines and antimicrobial peptides in the colonic tissue. (H) Representative immunohistochemical analysis of MUC2 in colonic tissue from WT and Muc2^△IEC^ mice. (I) Muc2^△IEC^ mice were administered water (control) or 4-GBA (150 mg/kg/d) orally for seven consecutive days, and stool samples were collected. qRT-PCR analysis of *A. muciniphila* abundance in feces. The data are the mean ± SD. Scale bar: 50 μm. *n* = 8 (B–F), *n* = 6 (G), *n* = 4 (H–I). Two-way ANOVA (B) or unpaired Student's *t*-test (C–G, I).

Given its established role as a probiotic in intestinal homeostasis,^23^^,^^24^ *A. muciniphila* was investigated as a potential mediator of 4-GBA's colitis amelioration. To determine whether 4-GBA's effects depend on this bacterium, the mice were pretreated with benzydamine hydrochloride (Ben) for two weeks to selectively deplete *A. muciniphila* before DSS-induced colitis and 4-GBA administration (Figure 4A). *In vitro* validation confirmed BEN's selective inhibition of *A. muciniphila* growth, while 16S rRNA sequencing revealed significant *in vivo* depletion of this species with minimal impact on other bacterial taxa (Figure S3A–C). Crucially, 4-GBA's protective effects were abolished in *A. muciniphila*-depleted mice (Figure 4B–G).

To elucidate the mechanism of 4-GBA's enrichment of *A. muciniphila*, we tested for direct effects of 4-GBA on this bacterium. *In vitro* growth assays demonstrated no stimulation of *A. muciniphila* by 4-GBA (Figure S3D). *Ex vivo* analysis of human and mouse stool-derived microbial communities further showed no significant alterations in overall bacterial composition or *Akkermansia* genus abundance following 4-GBA treatment (Figure S3E–J). These findings collectively indicate that 4-GBA does not directly modulate *A. muciniphila* abundance or gut microbiota composition in controlled environments.

Since *A. muciniphila* colonization is mucus-dependent,^25^ and 4-GBA enhances MUC2 production both *in vivo* and *ex vivo*. We hypothesized that mucus upregulation mediates 4-GBA's enrichment of *A. muciniphila*. To test this, we generated intestinal epithelium-specific Muc2 knockout mice (termed “Muc2^∆IEC^”) (Figure 4H). qPCR analysis revealed comparable *A. muciniphila* abundance between 4-GBA-treated and water-treated Muc2^∆IEC^ mice (Figure 4I), demonstrating that mucus production is essential for 4-GBA's enrichment of *A. muciniphila*.

### 3.5. 4-GBA enhances intestinal stem cell function via Hedgehog signaling activation

To further investigate 4-GBA's effects on mucosal barrier independently of the gut microbiota, we depleted the intestinal microbiota using antibiotics and assessed 4-GBA's effects on gene expression *in vivo*. 4-GBA exposure upregulated key ISCs markers (Lgr5, Ascl2, Sox9, and Mki67), goblet cell-associated markers (Muc2 and Tff3), and differentiation regulators (Atoh1 and Spdef) (Figure S4A and B). Consistently, 4-GBA treatment in antibiotic-treated mice also enhanced ISCs function, increased goblet cell numbers, and elevated MUC2 and OCCLUDIN expression, while enteroendocrine cell counts and apoptosis remained unaffected (Figure S4C–I). These results confirm that 4-GBA directly supports epithelial homeostasis independent of microbial interactions.

To validate this effect, we administered 4-GBA to colonic organoids derived from wild-type mice. The treated organoids showed significantly improved organoid-forming efficiency and increased budding capacity (Figure S4J). Gene expression analysis revealed the upregulation of ISCs-related markers, goblet cell markers and differentiation regulators in the 4-GBA-exposed organoids (Figure S4K). Enhanced proliferation was also noticed in the 4-GBA group (Figure S4L). Increased numbers of Lgr5-Tomato^+^ ISCs were observed in the 4-GBA-treated organoids derived from Lgr5-Tomato reporter mice (Figure S4M). These findings collectively establish that 4-GBA directly augments ISCs function independently of the microbiota.

**Figure 5.**
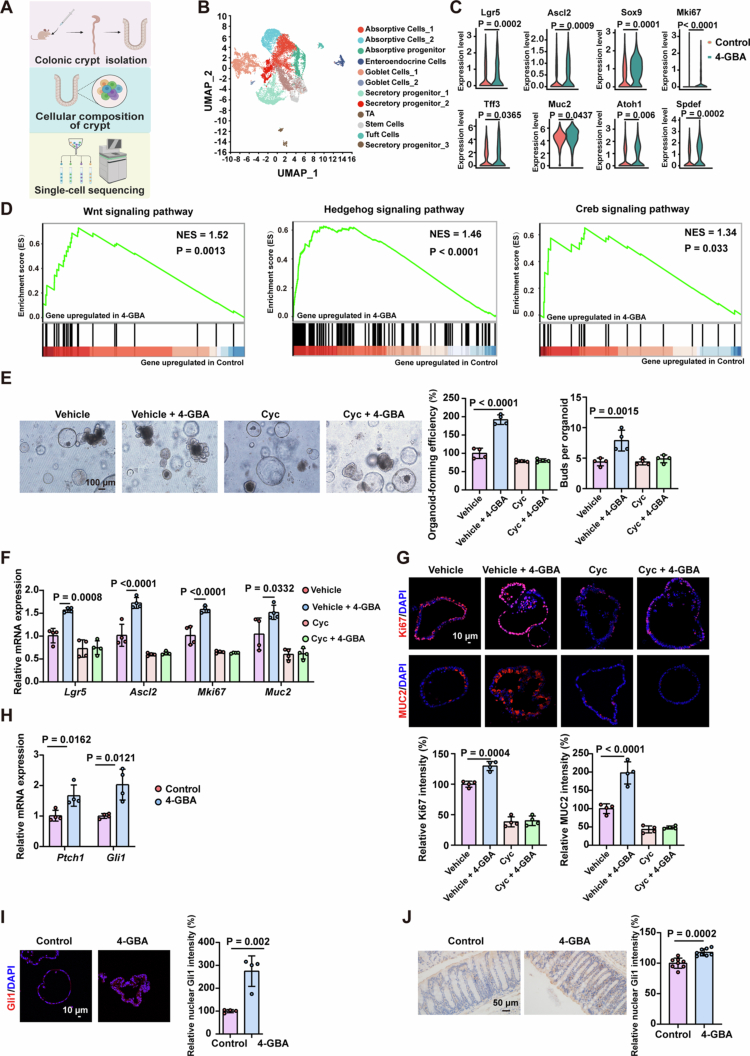
4-GBA enhances intestinal stem cell function through activation of the Hedgehog signaling pathway. (A–D) Colonic crypts were isolated from antibiotic-treated mice following exposure to 4-GBA or the water control and subjected to single-cell RNA sequencing analysis. (A) Schematic of scRNA-seq of colonic crypts. (B) UMAP plots of distinct clusters with scRNA-seq. (C) Violin plots depict the expression levels of stem cell-associated genes (Lgr5, Ascl2, Sox9, and Mki67) within the identified stem cell cluster and goblet cell-associated markers (Tff3, Muc2, Atoh1, and Spdef) within the goblet cell cluster. (D) Gene set enrichment analysis (GSEA) plots depicting enrichment of the Wnt signaling pathway, Hedgehog signaling pathway genes, and Creb signaling pathway-related genes within differentially expressed genes (DEGs) identified in the stem cell cluster comparing the 4-GBA-treated samples versus controls. (E–G) *Ex vivo* organoid formation of colonic crypts isolated from WT mice and exposed to 4-GBA (0.8 mM) in combination with or without cyclopamine (1 μM) for 4 d. (E) Representative images and quantification of organoid-forming efficiency and the numbers of buds per organoid. (F) mRNA expression levels of ISCs function markers (Lgr5, Ascl2, and Mki67) and a goblet cell markers (Muc2) in the indicated organoid. (G) Representative images and quantification of immunofluorescence staining against MUC2 and Ki67 in the indicated organoid. (H) mRNA expression levels of *Ptch1* and *Gli1* in the organoid exposed to 4-GBA or water control. (I) Representative images and quantification of immunofluorescence staining against Gli1 in the organoid exposed to 4-GBA or the water control. (J) Representative immunohistochemical analysis and quantitation of Gli1 in colonic tissue from antibiotic-treated mice following exposure to 4-GBA or the water control. The data are the mean ± SD. Scale bar: 10 μm, 50 μm, or 100 μm. *n* = 4 (E–I), *n* = 8 (J). Unpaired Student's *t*-test (C, H–J) or one-way ANOVA (E–G).

To identify the molecular mechanism, we performed single-cell RNA sequencing on colonic crypts from antibiotic-treated mice administered 4-GBA (Figure 5A). UMAP analysis resolved 12 distinct epithelial cell populations (Figures 5B and S5A). In the stem cell cluster, 4-GBA treatment significantly enhanced expression of ISCs function-related genes compared to controls (Figure 5C). Concurrently, goblet cell differentiation markers and mucus production genes were upregulated in the goblet cell cluster (Figure 5C). Pathway analysis of differentially expressed genes in the stem cell cluster revealed strong activation of the following pathways: cAMP, FoxO, Hedgehog, and Wnt (Figures 5D and S5B and C).

To functionally validate these pathways, we treated 4-GBA-exposed organoids with pathway-specific inhibitors: SQ22536 (cAMP), AS1842856 (FoxO), cyclopamine (Hedgehog), and IWP-2 (Wnt). Only Hedgehog pathway inhibition (cyclopamine) abrogated 4-GBA-induced effects, including the organoid-forming efficiency, budding capacity, and transcript levels of ISCs and goblet cell markers (Figure 5E and F), while these effects were not observed with cAMP, FoxO, or Wnt pathway inhibition (Figure S5D–I). Consistently, cyclopamine treatment abolished the 4-GBA-induced upregulation of Ki67 and MUC2 levels (Figure 5G).

Hedgehog pathway activation was characterized by the transcriptional upregulation of *Ptch1* and *Gli1* and enhanced nuclear translocation of the Gli1 protein. In colonic organoids-a pure epithelial system lacking mesenchymal cells-4-GBA treatment significantly increased *Ptch1* and *Gli1* mRNA levels (Figure 5H). Consistent with this, a marked increase in nuclear Gli1 was observed both in the organoids and specifically within the epithelial compartment of the colonic tissues following 4-GBA administration (Figure 5I and J). These data demonstrate that 4-GBA activates the Hedgehog pathway within intestinal epithelial cells. This epithelial-intrinsic activation is functionally essential, as Hedgehog inhibition completely abrogated the 4-GBA-mediated enhancement of ISCs function (Figure 5E–G). The concurrent observation of Gli1-positive stromal cells *in vivo* aligns with the established paracrine nature of intestinal Hedgehog signaling (Figure 5J), suggesting potential downstream epithelial‒mesenchymal crosstalk, while the core functional response originates from the epithelium.

### 3.6. 4-GBA modulates intestinal stem cell function via the SLC36A1–Hedgehog axis

**Figure 6.**
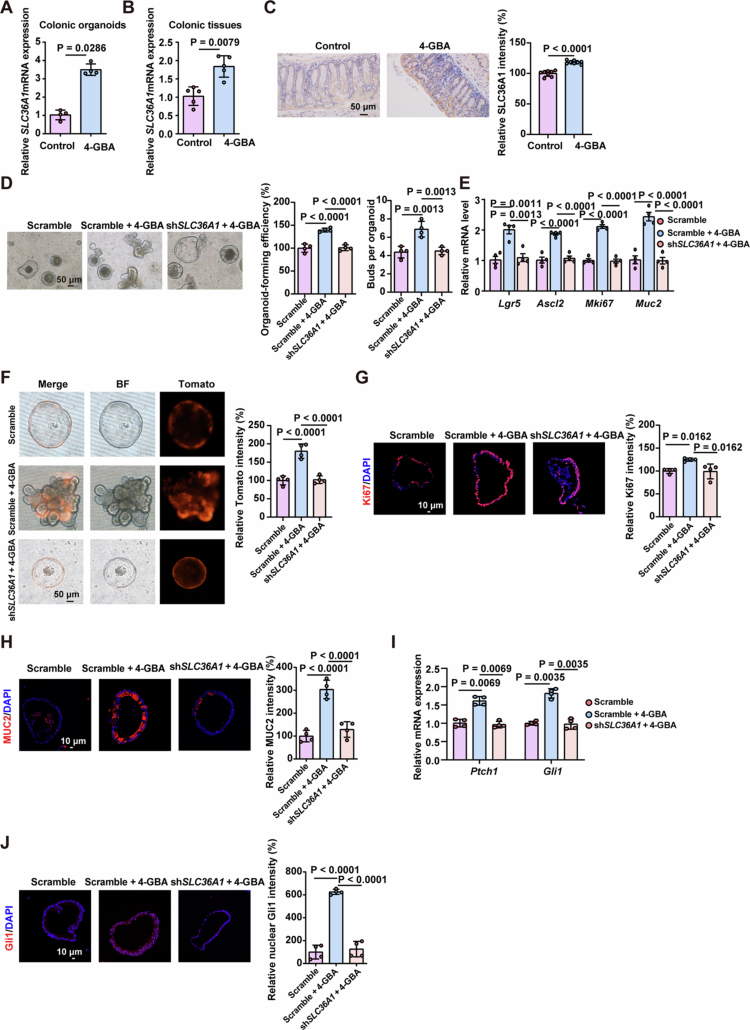
4-GBA activates intestinal stemness via SLC36A1–Hedgehog signaling. (A) mRNA expression levels of SLC36A1 in organoids exposed to 4-GBA or the water control. (B) mRNA expression levels of SLC36A1 in the colonic tissue from antibiotic-treated mice following exposure to 4-GBA or the water control. (C) Representative images and quantification of immunofluorescence staining against SLC36A1 in the colonic tissue from antibiotic-treated mice following exposure to 4-GBA or the water control. (D–J) Colonic organoid isolated from WT mice (D and E, G–J) or Lgr5-Tomato reporter mice (F) were infected with lentiviral vectors encoding either scramble control or SLC36A1-targeting shRNA for 48 h, followed by 4-d exposure to 4-GBA (0.8 mM) or water control. (D) Representative images and quantification of organoid-forming efficiency and the number of buds per organoid. (E) mRNA expression levels of ISCs function markers (Lgr5, Ascl2, and Mki67) and goblet cells markers (Muc2) in the indicated organoid. (F) Tamoxifen (2 μM) was administered on days 2–4 post treatment-initiation to label Lgr5-Tomato^+^ ISCs. Representative images and quantification of Lgr5-Tomato^+^ ISCs in the indicated organoid. (G and H) Representative images and quantification of immunofluorescence staining against Ki67 (G) and MUC2 (H) in the indicated organoid. (I) mRNA expression levels of *Ptch1* and *Gli1* in the indicated organoid. (J) Representative images and quantification of immunofluorescence staining against Gli1 in the indicated organoid. Data are the mean ± SD. Scale bar: 10 μm or 50 μm. *n* = 4 (A, D–J), *n* = 8 (B), *n* = 8 (C). Unpaired Student's *t-*test (A‒C) or one-way ANOVA (D‒J).

Given that 4-GBA is established as a substrate of the H⁺-coupled amino acid transporter SLC36A1,^21^ we investigated whether SLC36A1 mediates 4-GBA's regulation of ISCs function. 4-GBA treatment significantly upregulated SLC36A1 expression in both intestinal organoids and murine colonic tissue compared to controls (Figure 6A–C). Lentiviral shRNA-mediated SLC36A1 knockdown in organoids abrogated 4-GBA's effects on ISCs function, as evidenced by reduced organoid-forming efficiency, budding capacity, transcript levels of ISCs and goblet cell markers, decreased numbers of Lgr5-Tomato^+^ ISCs, and expression of Ki67 and MUC2 (Figure 6D–H). Notably, SLC36A1 knockdown also abolished 4-GBA-induced Hedgehog signaling activation, including reduced Ptch1 and Gli1 transcription and impairing the nuclear translocation of Gli1 in 4-GBA-treated organoids (Figure 6I and J). These findings demonstrate that SLC36A1 is essential for 4-GBA's enhancement of ISCs function.

To validate SLC36A1's role in 4-GBA-mediated epithelial homeostasis *in vivo*, we administered forskolin (a SLC36A1 inhibitor) to 4-GBA-treated mice (Figure S6A). While forskolin had no effect on histopathological changes or colon length (Figure S6B–C), it antagonized 4-GBA-driven epithelial homeostasis, as indicated by reduced numbers of Lgr5-Tomato^+^ ISCs and goblet cells, as well as decreased transcript levels of ISCs and goblet cell markers and diminished expression of Ki67, MUC2, and OCCLUDIN (Figure S6D–I). Furthermore, forskolin administration compromised 4-GBA-mediated activation of the Hedgehog pathway *in vivo* (Figure S6J and K). Interestingly, fecal *A. muciniphila* abundance was significantly reduced in the mice co-treated with forskolin and 4-GBA (Figure S6L). Collectively, these results demonstrate that SLC36A1 is required for 4-GBA's effects on epithelial homeostasis.

### 3.7. SLC36A1 upregulation mediates intestinal stem cell function and confers colitis resistance

**Figure 7.**
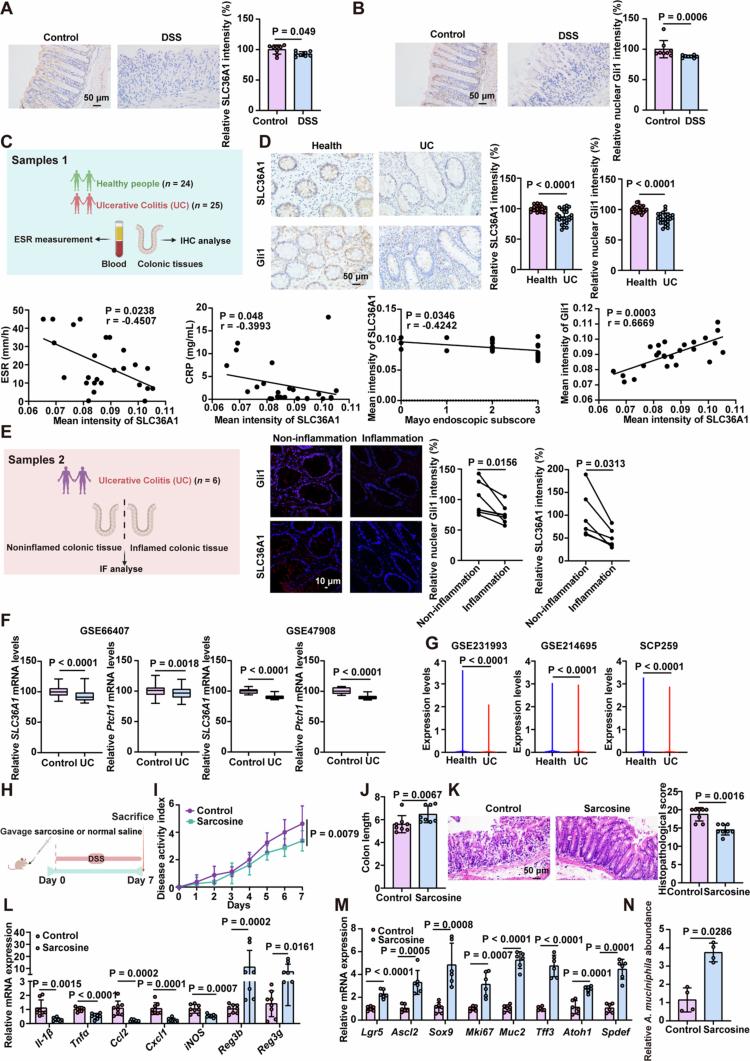
Regulation of SLC36A1 is associated with clinical colitis, and its activation ameliorates colitis progression. (A and B) Representative immunohistochemical analysis and quantitation of SLC36A1 (A) and Gli1 (B) in colonic tissue from DSS-treated mice or water control. (C) Diagram of the human samples. (D) Representative immunohistochemical analysis and quantitation of SLC36A1 and Gli1 in colonic tissue from healthy controls and colitis patients. Correlative analysis of SLC36A1 with the erythrocyte sedimentation rate (ESR), C-reactive protein (CRP), the Mayo endoscopic subscore, and Gli1 expression in colonic tissues. Correlations between variables were determined by linear regression analysis. The correlation coefficient (*r*) and associated *p*-value were calculated. (E) Diagram of the human samples (left). Representative images and quantification of immunofluorescence staining against SLC36A1 and Gli1 in non-inflamed and inflamed colonic regions from UC patients (right). (F) Box plot of *SLC36A1* and *Ptch1* mRNA in healthy controls and UC patients using publicly available NCBI GEO. (G) Violin plots of SLC36A1 gene expression in colonic epithelial cells using publicly available NCBI GEO and single-cell portal datasets. (H–L) Mice received intragastric pretreatment with sarcosine (500 mg/kg) followed by a 7-d challenge with 2% DSS in the drinking water. (H) Schematic representation of the experimental workflow. (I and J) Analysis of disease activity index scores (I) and colon length (J) of the indicated mice. (K) Representative H&E staining analysis and quantitation of histopathological changes in colonic tissue. (L) mRNA expression levels of proinflammatory cytokines and antimicrobial peptides in the colonic tissue. (M and N) Mice received a single intragastric dose of sarcosine (500 mg/kg) via oral gavage. Following pretreatment, the animals were maintained on standard drinking water for a 7-d observation period. Stool samples were collected. (M) mRNA expression levels of stem cell-associated genes (Lgr5, Ascl2, Sox9, and Mki67) and goblet cell-associated markers (Tff3, Muc2, Atoh1, and Spdef) in colonic tissue. (N) qRT-PCR analysis of *A. muciniphila* abundance in feces. The data are the mean ± SD. Scale bar: 10 μm or 50 μm. *n* = 8 (A and B, I–L), *n* = 24 or 25 (D), *n* = 6 (E, M), and *n* = 4 (N). Unpaired Student's *t*-test (A, B, D, F, G, J–N), two-way ANOVA (I) or paired Student's *t*-test (E).

To test the hypothesis that SLC36A1–Hedgehog axis activity contributes to mucosal barrier homeostasis and colitis progression, we first examined SLC36A1 expression in DSS-induced colitis models. Immunohistochemical analysis revealed significantly reduced SLC36A1 levels in the colonic tissues of colitis mice compared to healthy controls (Figure 7A). Parallel assessment of Hedgehog signaling activity confirmed its suppression in colitis tissues (Figure 7B).

We validated these findings in human samples using colonic biopsies from healthy controls and ulcerative colitis (UC) patients. Quantitative analysis demonstrated marked decreases in both SLC36A1 expression and Hedgehog signaling activity in UC tissues (Figure 7C and D). Notably, SLC36A1 expression exhibited a significant inverse correlation with clinical-inflammatory measures of UC severity (including ESR, CRP, and the Mayo endoscopic subscore), while showing a positive correlation with Gli1 nuclear translocation (Figure 7D). Analysis of paired non-inflamed and inflamed colonic regions from UC patients further revealed reduced SLC36A1 expression and nuclear Gli1 localization in inflamed tissues (Figure 7E). Multi-cohort validation using public bulk RNA sequencing datasets (GSE66407 and GSE47908) confirmed decreased SLC36A1 expression and Hedgehog signaling in patients with colitis compared to controls (Figure 7F). Moreover, single-cell RNA sequencing datasets (GSE231993, GSE214695; and SCP259) revealed dramatically decreased SLC36A1 expression in the colonic epithelial cells of colitis patients versus healthy controls (Figure 7G). These data establish a critical link between diminished SLC36A1 levels, suppressed Hedgehog signaling, and the severity of colitis.

To determine whether SLC36A1 modulation directly influences colitis outcomes, we engineered SLC36A1-overexpressing colonic organoids. SLC36A1 overexpression enhanced ISCs-driven phenotypes, including increased organoid-forming efficiency and budding capacity, elevated Lgr5-Tomato^+^ ISCs numbers and expression of ISCs and goblet cell markers, higher Ki67 and MUC2 expression (Figure S7A–E). Concurrently, Hedgehog signaling was robustly activated, as evidenced by upregulated Gli1 and Ptch1 transcripts and enhanced nuclear Gli1 translocation in SLC36A1-overexpressing organoids (Figure S7F and G).

To explore the therapeutic potential, we screened several substrates of SLC36A1, and identified sarcosine as a SLC36A1-inducing compound, and assessed its anti-inflammatory effects in DSS-induced colitis. Sarcosine treatment significantly increased SLC36A1 expression in murine colonic organoids and tissues, enhanced ISCs function, and activated Hedgehog signaling in murine colonic organoids (Figure S7H–P). *In vivo* studies demonstrated that sarcosine administration improved both the clinical and histopathological features of DSS-induced colitis in mice compared to untreated controls (Figure 7H–L). Moreover, increased expression of ISCs and goblet cell markers, as well as higher fecal abundance of *A. muciniphila*, were observed in the sarcosine-treated group without DSS treatment (Figure 7M and N).

Collectively, these findings demonstrate that SLC36A1 mediates intestinal stem cell function through Hedgehog signaling activation, sarcosine-induced SLC36A1 upregulation ameliorates colitis progression, and SLC36A1 represents a potential therapeutic target for colitis management.

## 4. Discussion

The interplay between the commensal microbiota, epithelial barriers, and mucosal secretions in the intestinal microenvironment critically drives the pathogenesis and progression of gut disorders.^9^ Targeting this triad is a key therapeutic strategy for restoring homeostasis. *Bacteroides*, a dominant gut genus, exerts significant effects on host physiology and microecology.^26-29^ Understanding its mechanisms via metabolites, microbial ecology, and epithelial interactions offers promise for uncovering disease pathways and developing interventions.

Using untargeted screening and metabolic profiling, we identified that the 4-GBA produced by *B. stercorirosoris* enhances ISCs functionality and stimulates goblet cell differentiation, ultimately alleviating colitis by enriching the intestinal niche for *A. muciniphila* (Figure S8). Our findings demonstrated that among the tested metabolites associated with arginine and proline metabolism, only 4-GBA induced *MUC2* expression and goblet cell differentiation in intestinal organoids. In contrast, capric acid, ectoine, agmatine, and 4-acetamidobutanoic acid failed to elicit comparable secretory activity across the same concentration range, supporting the conclusion of biological specificity. This functional selectivity probably stems from fundamental differences in physicochemical properties and transporter affinity. 4-GBA is a small, zwitterionic, guanidino-containing metabolite and a known substrate of the H⁺-coupled transporter SLC36A1, which exhibits structural differences from the other candidates.^30-35^ These results strongly suggest that the bioactivity is not a generic property of all arginine-derived metabolites but depends on specific structural features—likely the presence of both a terminal guanidino group (for target engagement) and a carboxylate moiety (for charge properties and transporter recognition), as uniquely exemplified by 4-GBA.

While prior studies report reduced *Bacteroidetes* and *A. muciniphila* in alcoholic heart disease^36^ and their synergistic antagonism of *Clostridioides difficile,*^37^ the mechanistic basis for their crosstalk remained unclear. Our work reveals a metabolite-driven axis where *Bacteroides*-derived 4-GBA modulates *A. muciniphila* abundance indirectly through host epithelial reprogramming, not direct microbial interaction. This redefines *Bacteroides-A. muciniphila* interactions as a host-dependent triad, suggesting that metabolite‒host‒microbe relays govern mucosal ecological patterning.

Prior studies report 4-GBA's role in gastric injury mitigation in rabbit models^19^ and as a biomarker in pulmonary fibrosis.^38^ However, our work identifies an epithelium-centric mechanism where microbiota–derived 4-GBA regulates intestinal homeostasis by directly modulating epithelial function to alleviate inflammation and enhance resistance to enteric pathogens. This finding redefines 4-GBA as a key mediator of the mucosal barrier through host-microbe signaling rather than a bystander metabolite.

This study demonstrated that 4-GBA upregulates SLC36A1 in intestinal epithelial cells, with its anti-colitis effects strictly dependent on this transporter. While SLC36A1 is known for transporting neutral amino acids (e.g., proline and glycine) and structural analogs (GABA and vigabatrin),^20^^,^^39^^,^^40^ its role in gut homeostasis was previously undefined. Clinically, a robust inverse correlation is observed between SLC36A1 expression and UC severity, with further suppression in inflamed versus non-inflamed regions. Analysis of available data from multi-cohort also demonstrated SLC36A1 expression is significantly reduced in UC patient compared to healthy controls, establishing reduced SLC36A1 expression as a potential diagnostic biomarker for UC. Mechanistically, SLC36A1 overexpression in murine organoids recapitulated 4-GBA's effects, enhancing ISCs activity and goblet cell differentiation. Notably, like other SLC36A1 substrates (e.g., taurine and betaine),^41-45^ sarcosine administration attenuated colitis progression *in vivo*. Given sarcosine's established safety as an adjunct therapy in neuropsychiatric disorders,^46^^,^^47^ our findings highlight its repurposing potential for UC treatment.

Our findings demonstrated that the treatment significantly elevated the fecal 4-GBA concentrations compared to vehicle controls. Importantly, the achieved concentration range exceeded the physiological levels of 4-GBA measured in fecal samples from healthy human donors (Figure S1M). This direct comparison indicates that our experimental approach establishes a pathophysiologically meaningful exposure within the intestinal lumen. It suggests that enhancing luminal 4-GBA, whether via direct supplementation or by modulation of its bacterial producers, could potentially represent a viable strategy for restoring mucosal homeostasis in colitis.

While our study employed pharmacological inhibition with forskolin to investigate SLC36A1 function *in vivo*, we recognize the interpretative limitations associated with this compound due to its pleiotropic effects as a cAMP activator. To strengthen causality, we complemented this approach with a specific genetic loss-of-function model *in vitro*, which demonstrated that SLC36A1 knockdown in intestinal organoids reduced the pro-mucogenic and stem cell-promoting effects of 4-GBA. Future studies will require the development of tissue-specific genetic models for SLC36A1 deletion to establish strong *in vivo* evidence of causality.

Notably, histological remission is increasingly recognized as a key therapeutic endpoint in UC. While the present study did not include systematic histological scoring, the strong inverse correlation we observed between SLC36A1 expression and both endoscopic severity and systemic inflammatory markers-coupled with its further suppression in inflamed tissues-strongly suggests that impairment of this pathway is closely linked to mucosal inflammatory injury. Therefore, restoring epithelial homeostasis via the 4-GBA/SLC36A1/Hedgehog axis may not only improve clinical–endoscopic outcomes but also provide a molecular foundation for achieving histological remission. Future prospective studies that directly assess this pathway alongside standardized histological scoring will be valuable to validate this hypothesis and to explore targeting this axis for attaining “deep remission” in UC.

Our work identifies *B. stercorirosoris* as the first characterized gut commensal producing 4-GBA, though its biosynthetic pathway and enzyme specificity remain undefined. The role of the intestinal microbiome in host 4-GBA exposure remains incompletely understood, with unknown contributions from other commensals/pathobionts. Systematic screening of dominant anaerobes and mucosa-associated taxa for 4-GBA production capacity is able to clarify this microbial metabolite‒host interplay in further studies.

Our study reveals 4-GBA's cell-type-selective targeting of colonic epithelia, correlating with SLC36A1's epithelium-restricted expression and implying receptor-mediated sensing. While its epithelial-specific effects on barrier function and mucosal homeostasis are evident, the compound's broader role in colonic communication remains unclear. Investigating 4-GBA's direct/indirect effects on non-epithelial compartments during colitis via alternative receptors or epithelial-immune signaling could further elucidate 4-GBA-mediated microbial-host crosstalk mechanisms.

Overall, our study identified a novel microbe‒host axis where *B. stercorirosoris*-derived 4-GBA strengthens the intestinal mucosal barrier via SLC36A1-dependent epithelial reprogramming. Importantly, SLC36A1 is demonstrated as a potential clinically relevant biomarker and therapeutic target for UC. Our findings reveal mechanistic insights into co-evolutionary dynamics between the gut microbiota and host mucosal systems, offering strategies to modulate microbiota‒epithelial interactions in intestinal inflammation.

## Supplementary Material

Supplementary material sub 20260206.pdf

## Data Availability

Single-cell RNA sequencing data and 16S rRNA gene sequencing data are available in the NCBI BioProject databases under the accession numbers PRJNA1298487, PRJNA1298490, and PRJNA1298199. The non-targeted metabolomics raw data are available through the Metabolomics Workbench project ID PR002560 (http://dx.doi.org/10.21228/M8R24X).
