# Supplementary material for: A 4-guanidinobutanoic acid-SLC36A1 axis drives a microbiota‒host feedback loop to regulate intestinal homeostasis
Source: Gut Microbes. 2026 Mar 4;18(1):2639216. doi: 10.1080/19490976.2026.2639216 (PMC12969751; doi:10.1080/19490976.2026.2639216)
Supplement: Supplementary material — sub 20260206.pdf [file KGMI_A_2639216_SM6810.pdf]

Supplementary materials

A 4-guanidinobutanoic acid-SLC36A1 axis drives a microbiota-host feedback loop to regulate intestinal homeostasis

Jianming Yang#, Yawen Xiao#, Jifang Cui#, Ruofan Song#, Wanxia Ma, Jiangpeng Liu, Chunhui Miao, Xinyu Sun, Xueting Kong, Zhi-Song Zhang, Lu Zhou, Zhi Yao, Quan Wang*

***Corresponding Author:**

Quan Wang

Email: wangquan@pumc.edu.cn (Q. W.)

This file includes:

Supplementary Figure S1 to S8

Supplementary Table S1-S2

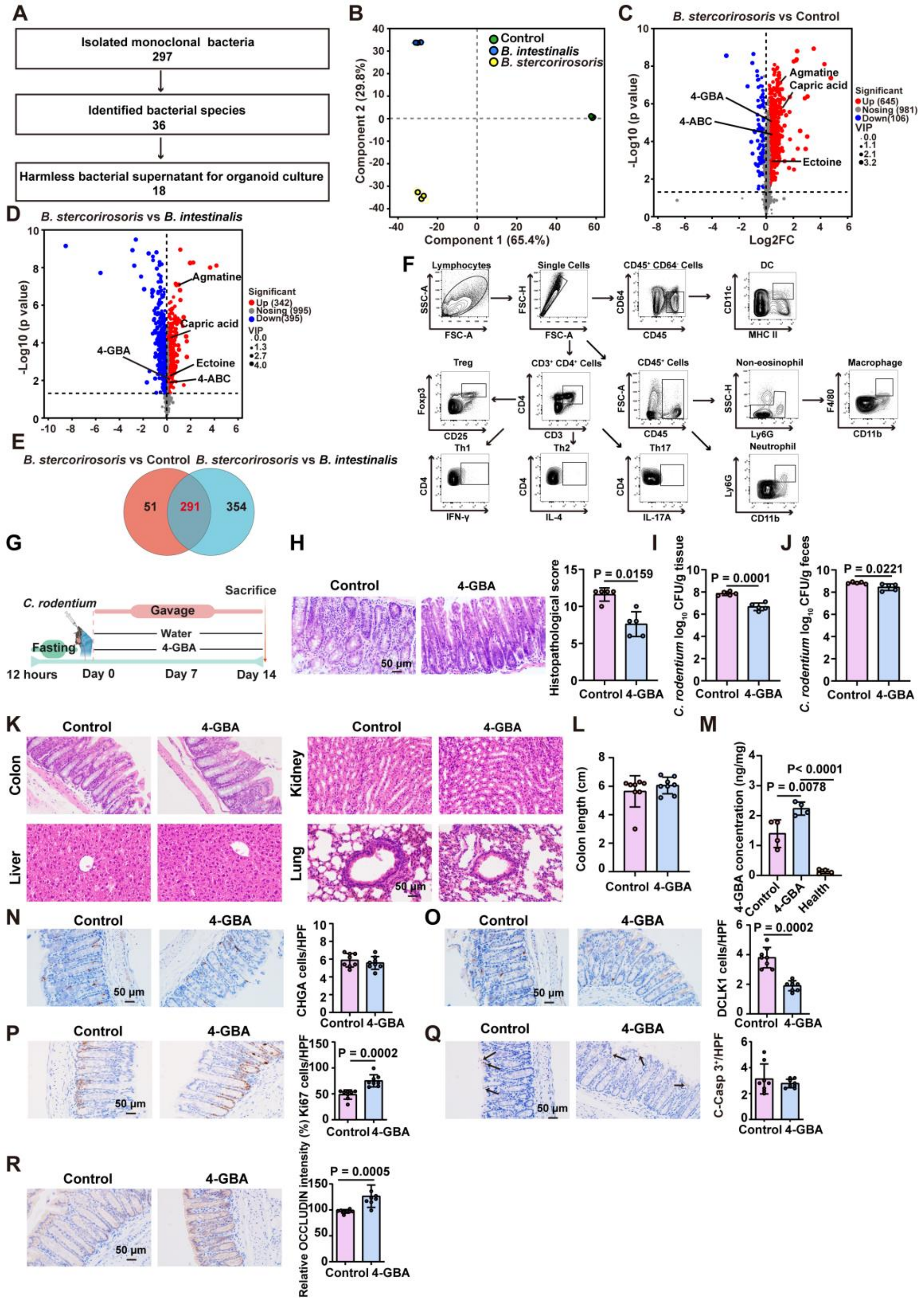

Figure S1. 4-GBA ameliorates *C. rodentium*-induced colitis. (A) Schematic of screening for commensal bacterial culture supernatants from fecal samples of healthy volunteers. **(B-E)** Untargeted metabolomics analysis of *B. stercorirosoris* supernatant using blank organoid medium and supernatant from the negative control strain *B.*

intestinalis as controls. **(B)** Principal component analysis (PCA) of the three groups. **(C)** Volcano plot showing the upregulated and downregulated metabolites in *B. stercorirosoris* supernatant versus blank medium. **(D)** Volcano plot showing the upregulated and downregulated metabolites in *B. stercorirosoris* supernatant versus *B. intestinalis* supernatant. **(E)** Venn diagram showing the 291 metabolites overlapped between these differential sets. **(F)** Gating strategy for flow cytometry analysis of immune cells. **(G-J)** Mice received intragastric pretreatment with 1 × 10^9 CFU *C. rodentium,* followed by a 14-day challenge with 4-GBA (150 mg/kg). **(G)** Schematic of the *C. rodentium*-induced colitis. **(H)** Representative H&E staining analysis and quantitation of the colonic pathology score. **(I-J)** *C. rodentium* colonization in colonic tissue (I) and feces (J) of control and 4-GBA-treated mice. **(K-R)** WT mice received daily oral gavage of 4-GBA (150 mg/kg) for 7 days. **(K)** Representative H&E staining of the colon, liver, lung, and kidney tissues. **(L)** Quantification of the colon length from the indicated mice. **(M)** Targeted metabolomics analysis of fecal 4-GBA concentrations in vehicle-treated control mice, 4-GBA-treated mice, and healthy human volunteers. **(N-R)** Representative CHGA (N), DCLK1 (O), Ki67 (P), cleaved caspase-3 staining (Q), and OCCLUDIN (R) and quantitation in colon sections from control and 4-GBA-exposed mice. Data are the mean ± SD. Scale bar: 50 μm. n = 5 (H-J), n = 4 (M), n = 8 (L, N-R). Unpaired Student's t-test (H-J, L, N-R) or one-way ANOVA (M).

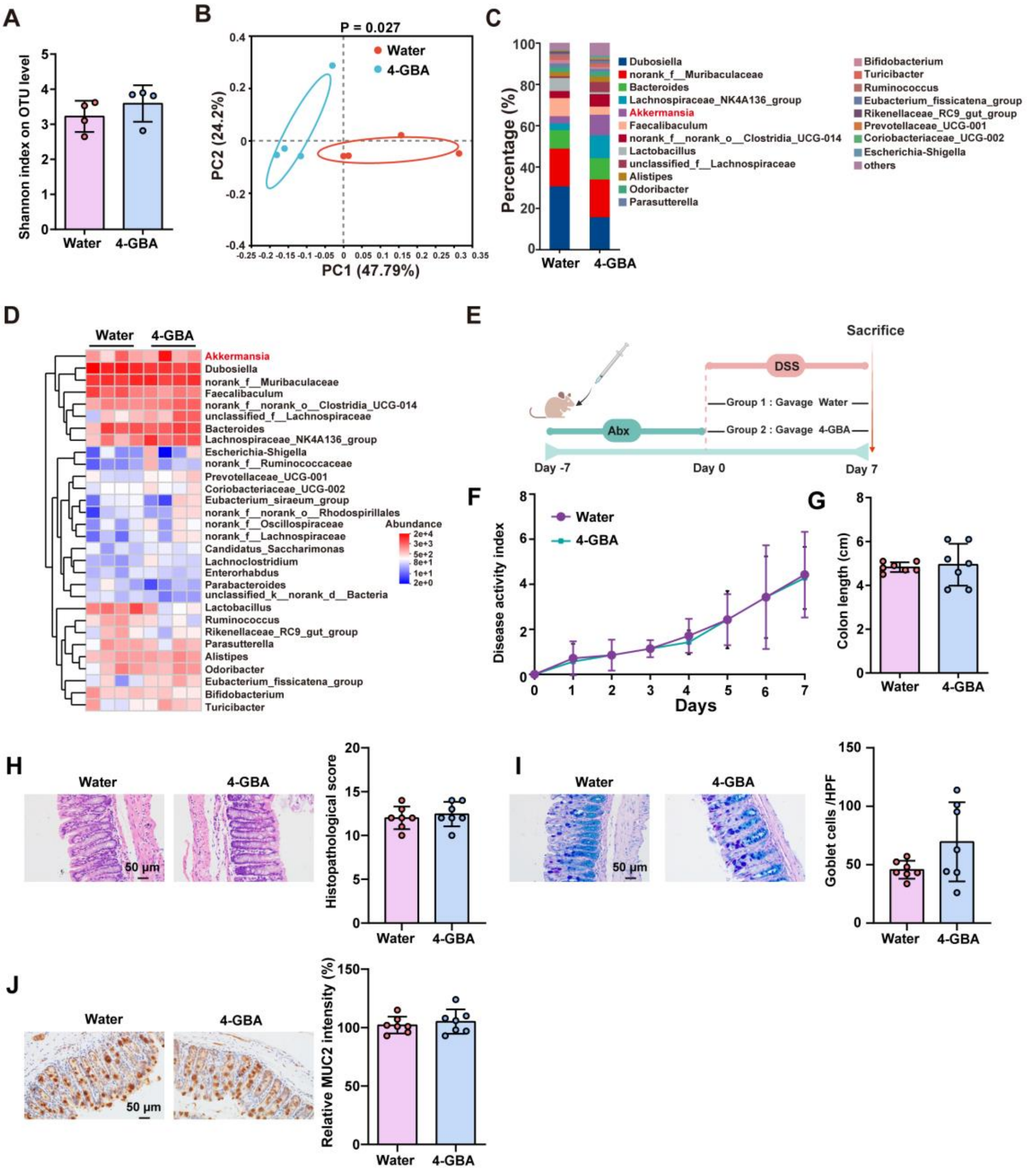

Figure S2. Gut microbiota mediate 4-GBA's therapeutic effects in colitis. (A-D) Mice received 2% DSS in drinking water with co-administration of 4-GBA (150 mg/kg) for 7 days. Stool samples from these mice were collected and analyzed by 16S rRNA gene sequencing. **(A)** Shannon index of the gut microbiota. **(B)** Principal component analysis of the fecal microbiota. **(C)** Percent of bacterial community abundance at the genus level. **(D)** Heatmap showing TOP 30 bacteria at the genus level. **(E-I)** Antibiotic-pretreated mice received 2% DSS challenge with co-administration of 4-GBA (150 mg/kg) for 7 days. **(E)** The schematic of gut microbiota depletion experimental design. **(F-J)** Analysis of disease activity index

scores (F) and colon length (G) of the indicated mice. **(H)** Representative H&E staining analysis and quantitation of histopathological changes in colonic tissue. **(I)** AB-PAS staining and quantitation of colonic tissue. **(J)** Representative immunohistochemical analysis and quantitation of MUC2 in colonic tissue. Data are the mean ± SD. Scale bar: 50 μm. n = 4 (A-D), n = 7 (F-J). Two-way ANOVA (F) or unpaired Student's t-test (A, G-J).

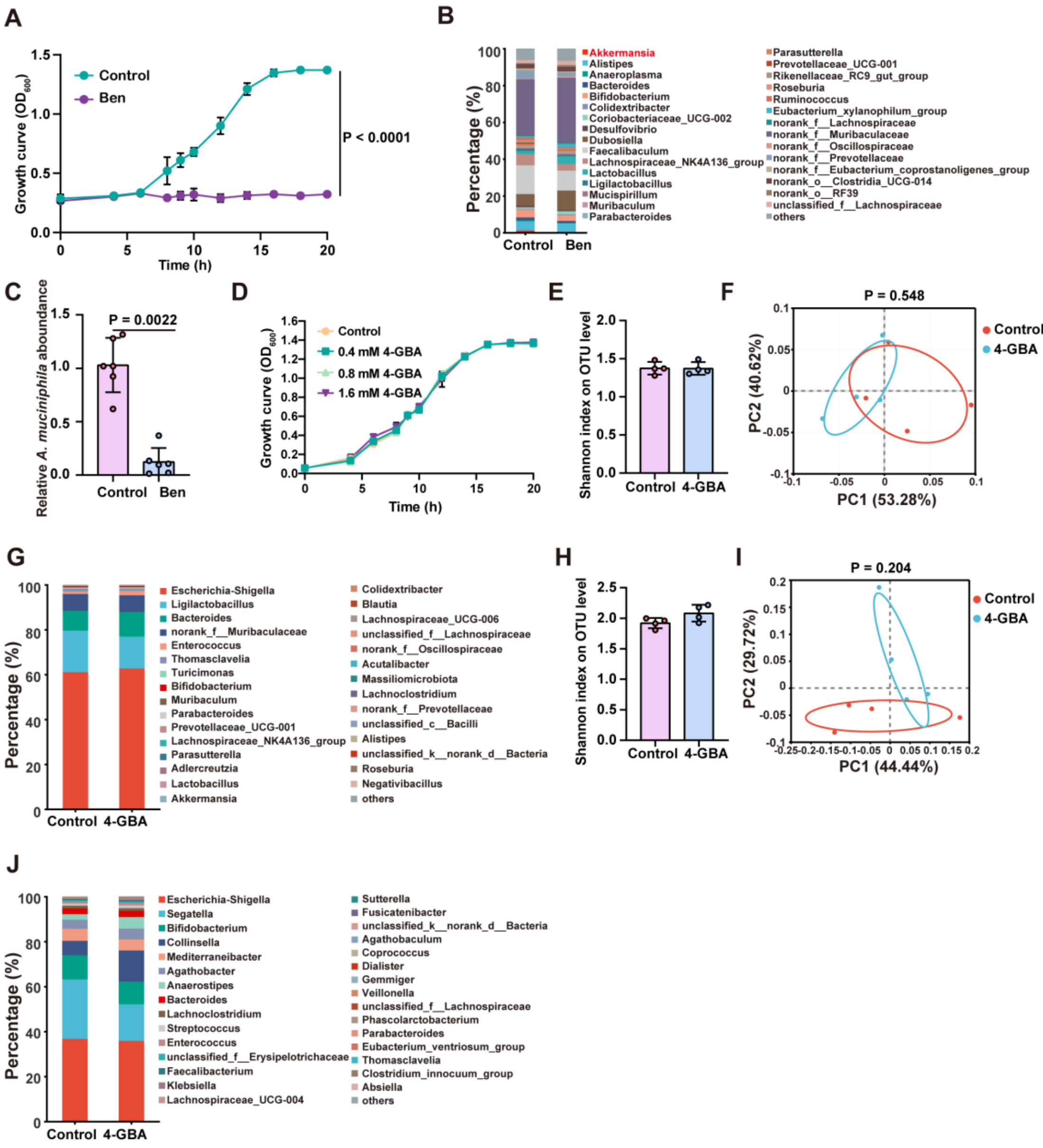

Figure S3. 4-GBA administration does not directly alter *A. muciniphila* abundance and overall gut microbiome composition. (A) The growth curve of the *A. muciniphila* cultured in BHI media supplemented with Ben benzydamine hydrochloride (50 µM) or PBS control anaerobically for 20 h. **(B-C)** Stool samples from benzydamine hydrochloride-treated mice (50 mg/kg) or PBS control were collected and analyzed by 16S rRNA gene sequencing analysis and *A. muciniphila* abundance analysis. **(B)** Percent of bacterial community abundance at the genus level. **(C)** qRT-PCR analysis of *A. muciniphila* abundance in feces. **(D)** The growth curve of the *A. muciniphila* anaerobically cultured in BHI media supplemented with 4-GBA at different concentrations or PBS control. **(E-J)** Mouse (E-G) and human (H-J)

stool-derived *ex vivo* microbial communities were cultured in mGAM media supplemented with 4-GBA (0.8 mM) or PBS control anaerobically for 48 h, followed by 16S rRNA gene sequencing analysis. **(E, H)** Shannon index of stool-derived *ex vivo* microbial communities. **(F, I)** Principal component analysis of stool-derived *ex vivo* microbial communities. **(G, J)** Genus-level compositions of stool-derived *ex vivo* microbial communities. Data are the mean ± SD. n = 3 (A, D), n = 6 (C), n = 4 (E-J). Two-way ANOVA (A, D) or unpaired Student's t-test (C, E, H).

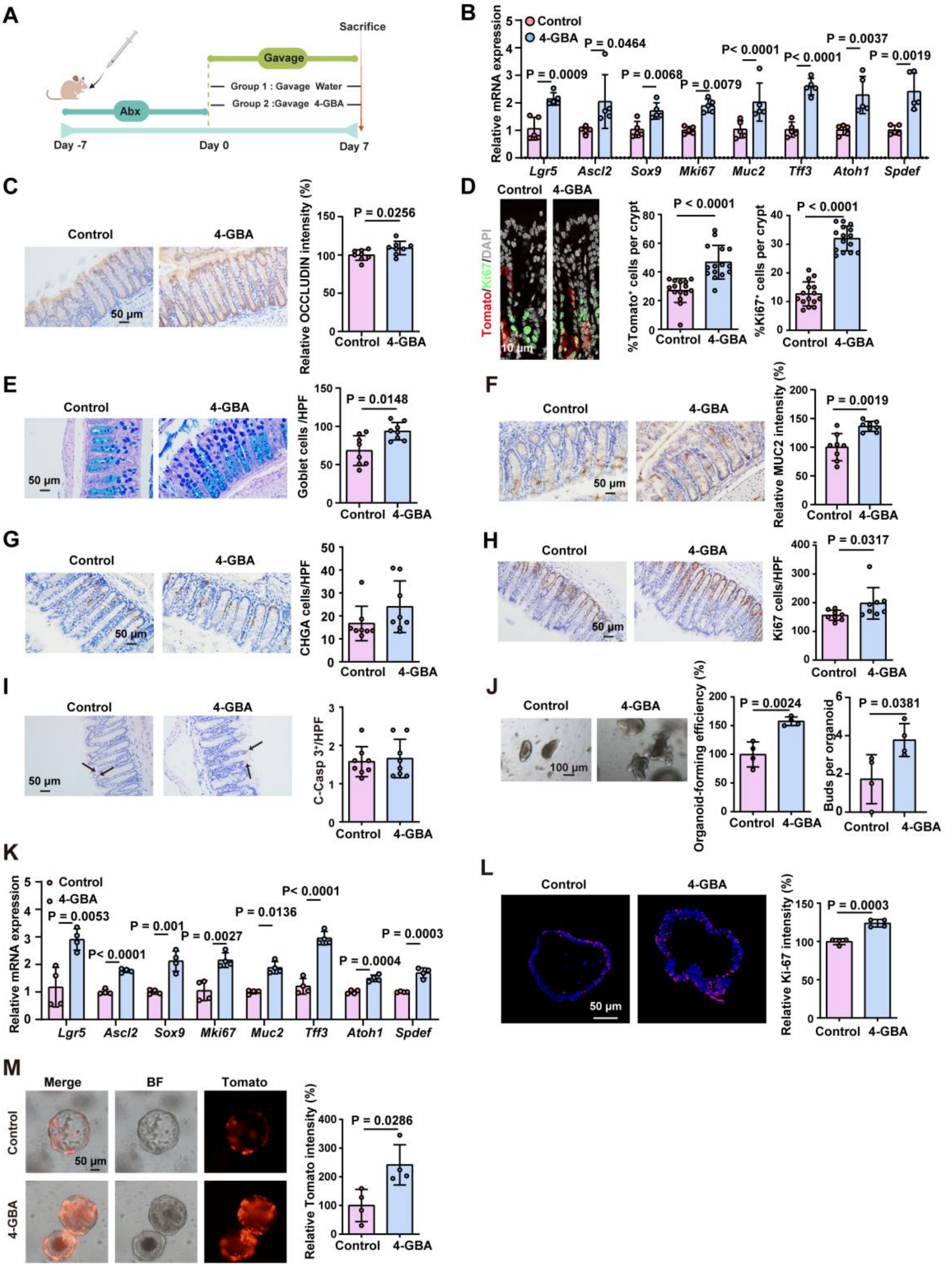

Figure S4. 4-GBA enhances mucosal barrier independently of gut microbiota. (A-J) WT mice (B-C, E-J) or Lgr5-Tomato reporter mice (D) were treated with antibiotic to deplete the intestinal microbiota and then were exposed to 4-GBA (150 mg/kg) or water control for 7 days. **(A)** Schematic of the workflow. **(B)** mRNA expression levels of stem cell-associated genes (Lgr5, Ascl2, Sox9, Mki67) and goblet cell-associated markers (Tff3, Muc2, Atoh1, Spdef) in colonic tissue. **(C)**

Representative immunohistochemical analysis and quantitation of OCCLUDIN in colonic tissue. **(D)** Tamoxifen (20 mg/kg/day, i.p.) was administered on days 5-7 to label Lgr5-Tomato$^+$ cells. Representative images and quantification of the percentages of Tomato$^+$ crypts and Ki-67$^+$ cells among total crypts in mouse colon tissue. **(E)** AB-PAS staining and quantitation of colonic tissue. **(F-I)** Representative MUC2 (F), CHGA (G), Ki67 (H), and cleaved caspase-3 (I) staining and quantitation in colon sections. **(J-M)** Colonic organoids were isolated from WT mice (J-L) or Lgr5-Tomato reporter mice (M) and then exposed to 4-GBA (0.8 mM) or water control for 4 days. **(J)** Representative images and quantification of organoid-forming efficiency and the numbers of buds per organoid. **(K)** mRNA expression levels of stem cell-associated genes (Lgr5, Ascl2, Sox9, Mki67) and goblet cell-associated markers (Tff3, Muc2, Atoh1, Spdef) in the indicated organoid. **(L)** Representative images and quantification of immunofluorescence staining against Ki67 in the indicated organoid. **(M)** Tamoxifen (2 μM) was administered on days 2-4 post treatment-initiation to label Lgr5-Tomato$^+$ ISCs. Representative images and quantification of Lgr5-Tomato$^+$ ISCs in the indicated organoid. Data are the mean ± SD. Scale bar: 10 μm, 50 μm, or 100 μm. n = 5 (B), n = 8 (C-I), n = 4 (J-M). Unpaired Student's t-test (B-M).

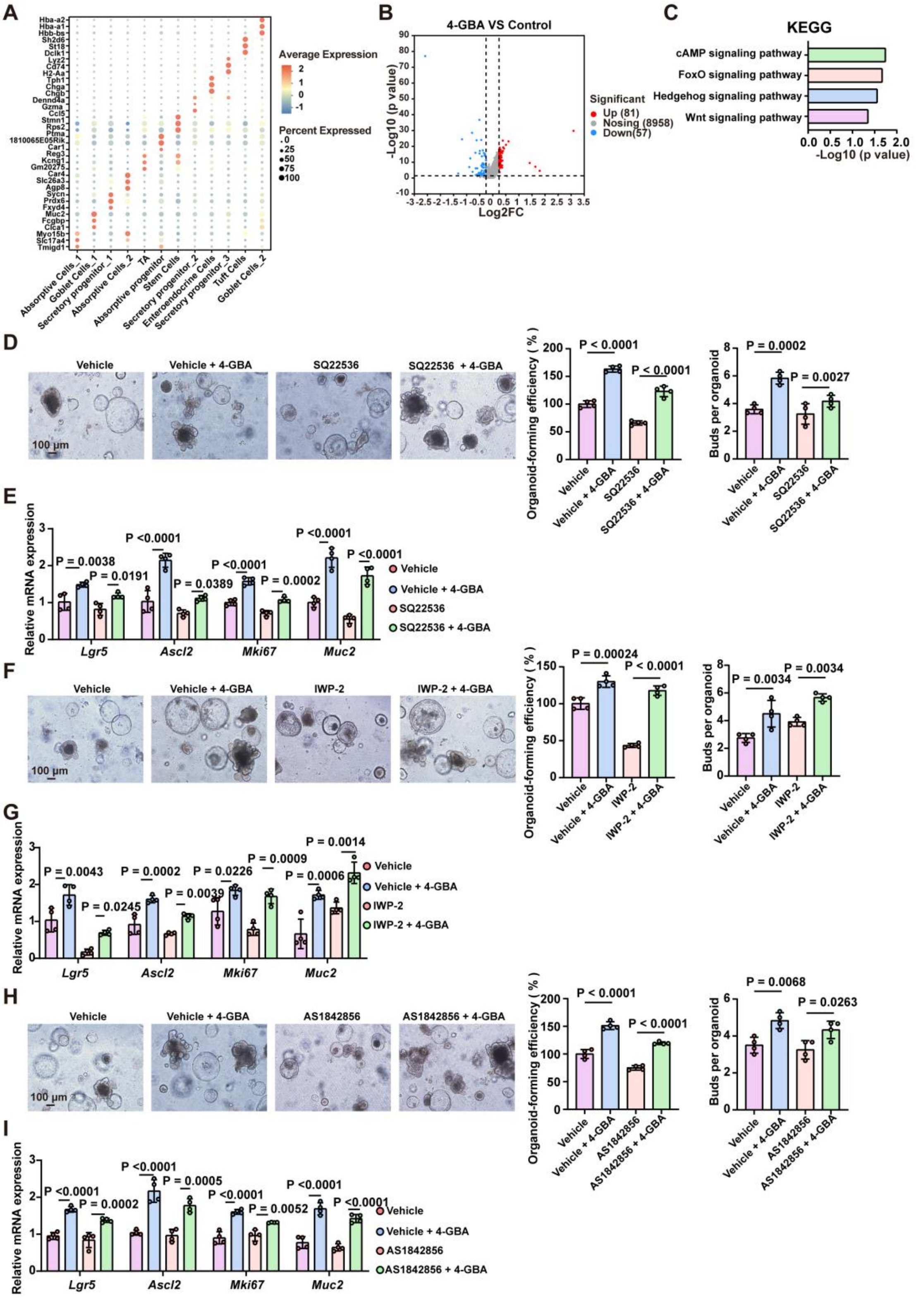

Figure S5. 4-GBA enhances intestinal stem cell function independently of cAMP, FoxO, and Wnt signaling pathways. (A-C) Colonic crypts were isolated from antibiotic-treated mice following exposure to 4-GBA or water control and subjected to single-cell RNA sequencing analysis. **(A)** Heatmap of top differential genes from

distinct clusters. **(B)** Volcano plot depicting differentially expressed genes (DEGs) identified in the stem cell cluster comparing 4-GBA-treated samples versus controls. **(C)** KEGG analysis depicting enrichment in DEGs overexpressed in the stem cell cluster comparing 4-GBA-treated samples versus controls. **(D-I)** Colonic organoids were isolated from WT mice and exposed to 4-GBA (0.8 mM) in combination with or without SQ22536 (100 μM), AS1842856 (1 μM), and IWP-2 (20 μM) for 4 days. **(D, F, H)** Representative images and quantification of organoid-forming efficiency and the numbers of buds per organoid. **(E, G, I)** mRNA expression levels of ISCs function markers (Lgr5, Ascl2, Mki67) and goblet cell markers (Muc2) in the indicated organoid. Data are the mean ± SD. Scale bar: 100 μm. $n = 4$ (D-I). One-way ANOVA (D-I).

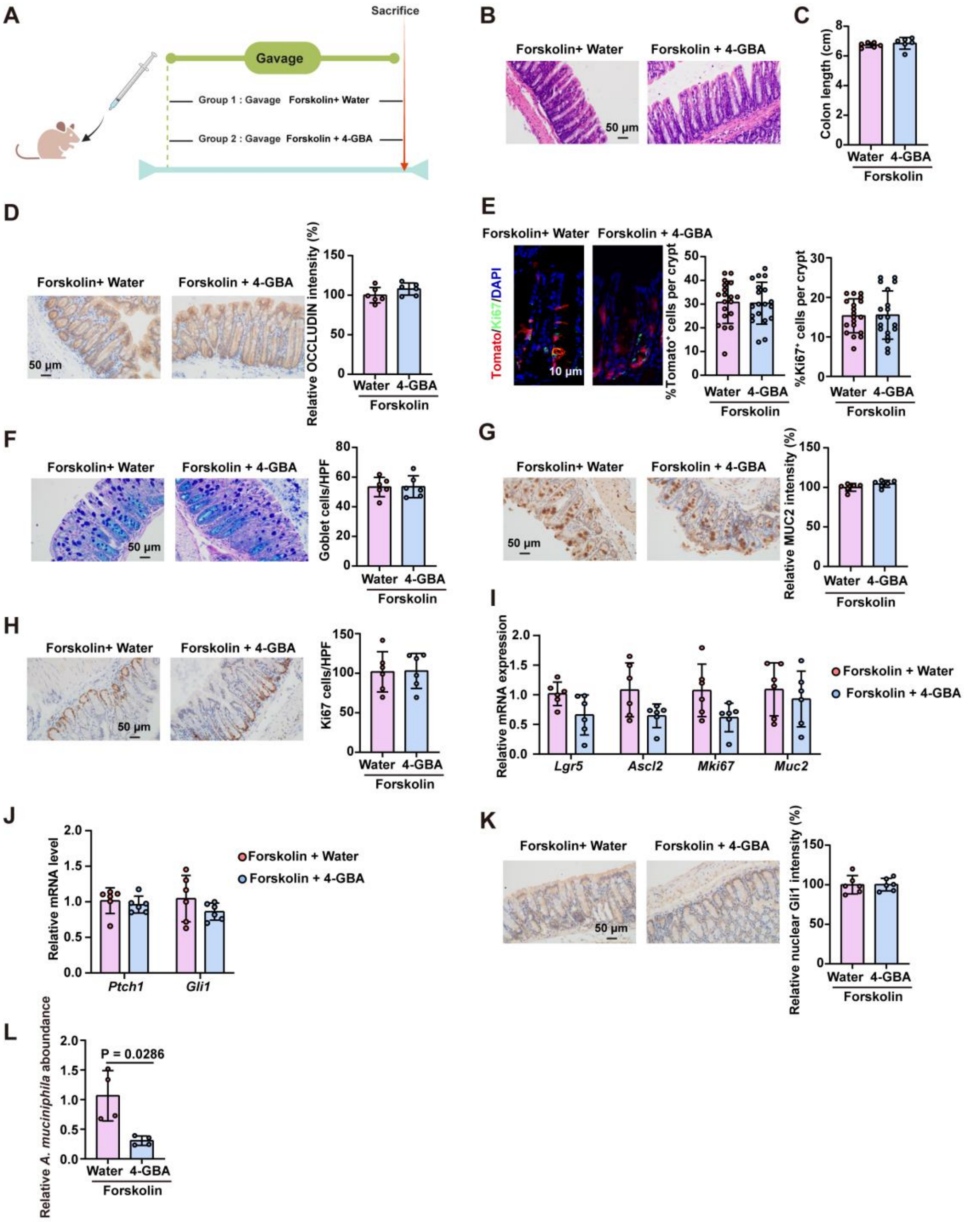

Figure S6. SLC36A1 inhibitor antagonizes 4-GBA-driven epithelial homeostasis. (A-L) WT mice (B-D, F-L) or Lgr5-Tomato reporter mice (E) received daily oral administration of 4-GBA (150 mg/kg) or water control for 7 days, with concurrent intraperitoneal Forskolin (100 mg/kg/day). **(A)** Schematic of the workflow. **(B)** Representative H&E staining analysis and quantitation of histopathological changes in colonic tissue. **(C)** Quantification of the colon length from the indicated mice. **(D)** Representative immunohistochemical analysis and quantitation of OCCLUDIN in

colonic tissue. **(E)** Tamoxifen (20 mg/kg/day, i.p.) was administered on days 5-7 to label Lgr5-Tomato^{+} cells. Representative images and quantification of the percentages of Tomato^{+} crypts and Ki-67^{+} cells among total crypts in mouse colon tissue. **(F)** AB-PAS staining and quantitation of colonic tissue. **(G-H)** Representative MUC2 (G) and Ki67 (H) staining and quantitation in colon sections. **(I)** mRNA expression levels of stem cell-associated genes (Lgr5, Ascl2, Sox9, Mki67) and goblet cell-associated markers (Tff3, Muc2, Atoh1, Spdef) in colonic tissue. **(J)** mRNA expression levels of *Ptch1* and *Gli1* in colonic tissue. **(K)** Representative Gli1 staining and quantitation in colon sections. **(L)** qRT-PCR analysis of *A. muciniphila* abundance in feces. Data are the mean ± SD. Scale bar: 10 μm or 50 μm. n = 6 (A-K). Unpaired Student's t-test (B-K).

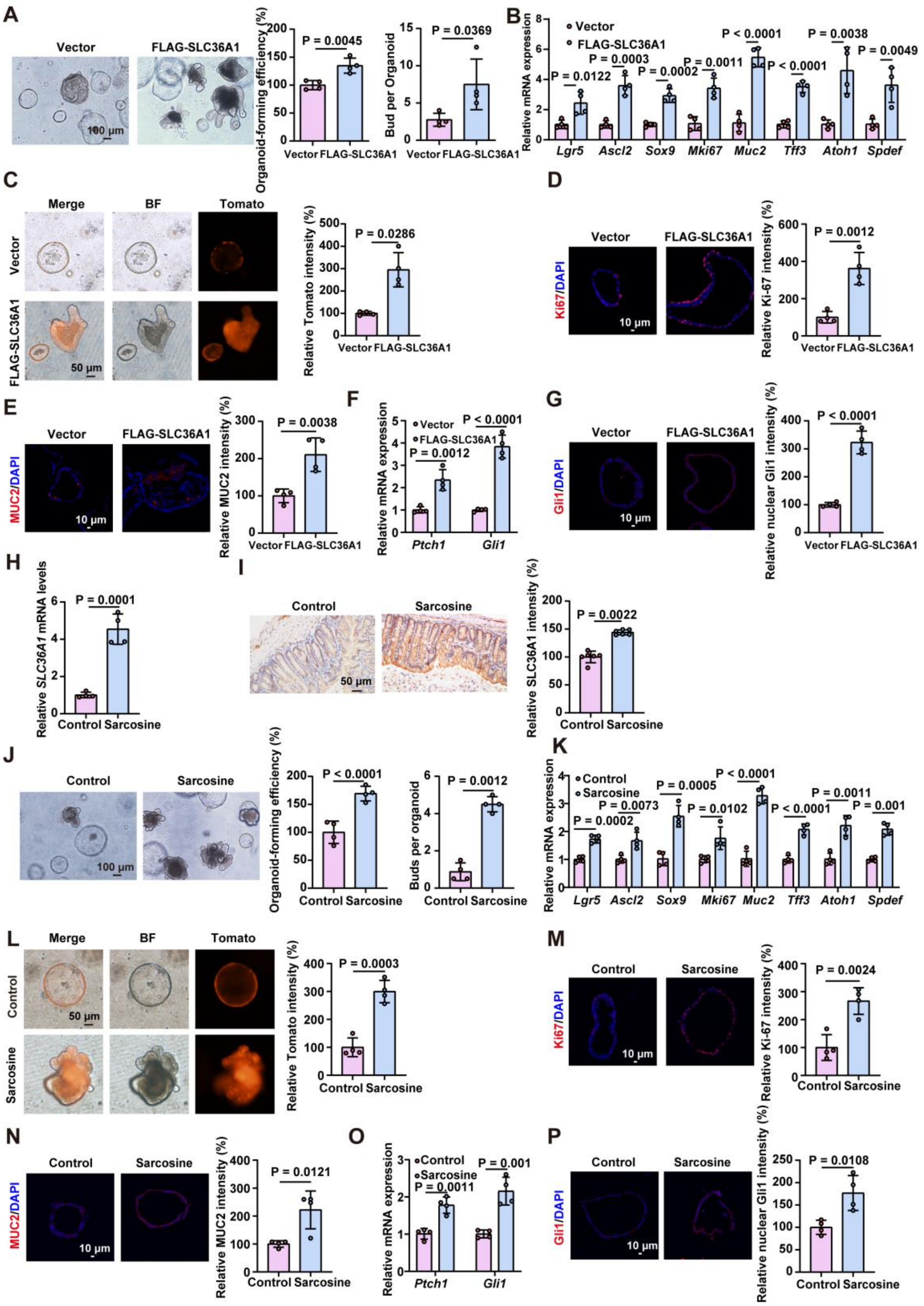

Figure S7. Sarcosine ameliorates colitis through up-regulating SLC36A1. (A-G) Colonic organoids isolated from WT mice (A-B, D-G) or Lgr5-Tomato reporter mice (C) were infected with lentiviral vectors encoding either vector control or FLAG-PAT1 for 48 hours. **(A)** Representative images and quantification of

organoid-forming efficiency and the numbers of buds per organoid. **(B)** mRNA expression levels of ISCs function markers and goblet cell markers in the indicated organoid. **(C)** Tamoxifen (2 μM) was administered for 3 days to label Lgr5-Tomato$^+$ ISCs. Representative images and quantification of Lgr5-Tomato$^+$ ISCs in the indicated organoid. **(D-E, G)** Representative images and quantification of immunofluorescence staining against Ki67 (D), MUC2 (E), and Gli1 (G) in the indicated organoid. **(F)** mRNA expression levels of *Ptch1* and *Gli1* in the indicated organoid. **(H)** mRNA expression levels of SLC36A1 in the organoid exposed to sarcosine (1 μM) or water control for 4 days. **(I)** Representative immunohistochemical analysis and quantitation of SLC36A1 in colonic tissue from sarcosine-exposed mice. **(J-P)** Colonic organoids were isolated from WT mice (J-K, M-P) or Lgr5-Tomato reporter mice (L) and then exposed to sarcosine (1 μM) or water control for 4 days. **(J)** Representative images and quantification of organoid-forming efficiency and the numbers of buds per organoid. **(K)** mRNA expression levels of stem cell-associated genes and goblet cell-associated markers in the indicated organoid. **(L)** Tamoxifen (2 μM) was administered on days 2-4 post treatment-initiation to label Lgr5-Tomato$^+$ ISCs. Representative images and quantification of Lgr5-Tomato$^+$ ISCs in the indicated organoid. **(M-N, P)** Representative images and quantification of immunofluorescence staining against Ki67 (M), MUC2 (N), and Gli1 (P) in the indicated organoid. **(Q)** mRNA expression levels of *Ptch1* and *Gli1* in the indicated organoid. Data are the mean ± SD. Scale bar: 10 μm, 50 μm, or 100 μm. *n* = 4 (A-H, J-P), *n* = 6 (I). Unpaired Student's t-test (A-N).

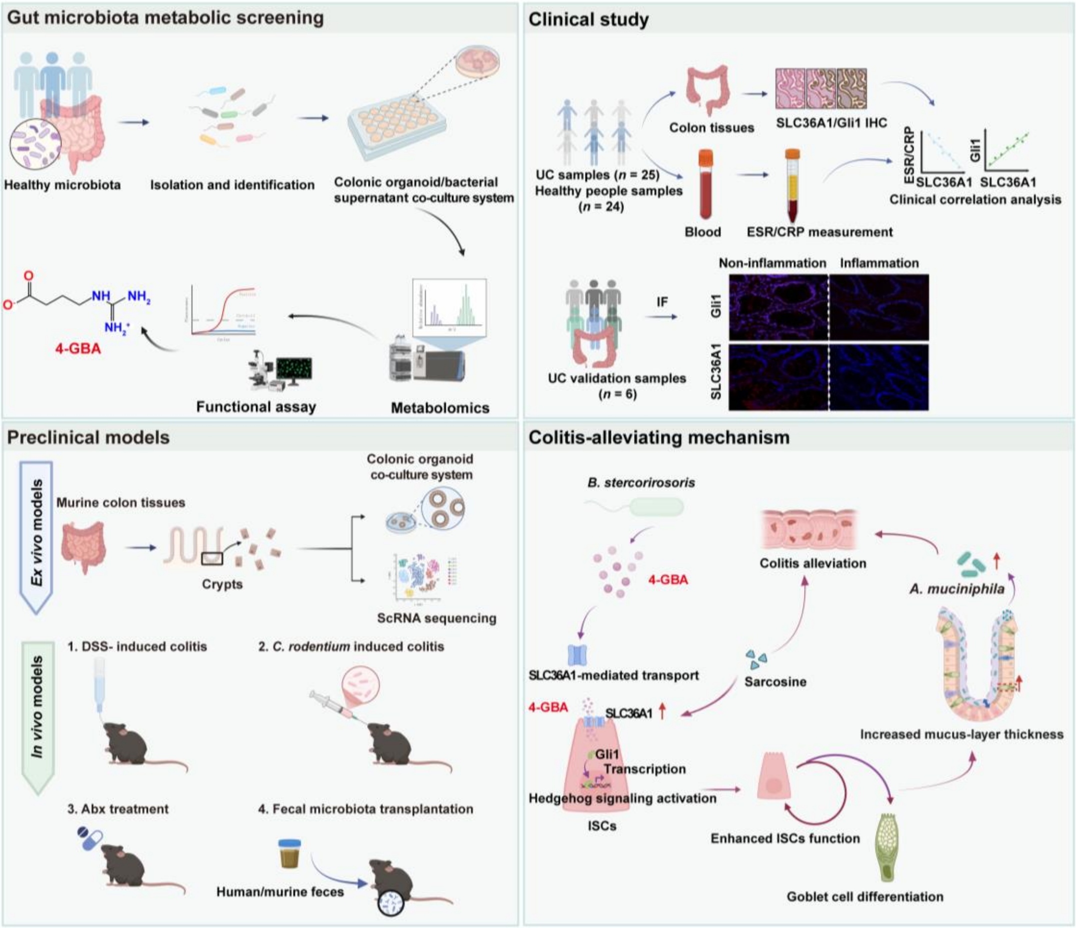

Figure S8. Graphical abstract illustrating the role of gut commensal 4-GBA in intestinal homeostasis. Schematic diagrams were generated by BioRender.com.

Table S1. Characteristics of healthy controls and patients with UC

Participants	Age (year)	Sex	Biopsy locations	Montreal classification	Mayo endoscopic subscore	ESR (mm/h)	CRP (mg/mL)	Medications	Note
1	39	female	Proximal colon	-	-	-	-	-	Healthy
2	67	male	Distal colon	-	-	-	-	-	Healthy
3	55	male	Distal colon	-	-	-	-	-	Healthy
4	50	male	Distal colon	-	-	-	-	-	Healthy
5	65	male	Distal colon	-	-	-	-	-	Healthy
6	56	male	Distal colon	-	-	-	-	-	Healthy
7	75	female	Distal colon	-	-	-	-	-	Healthy
8	58	female	Distal colon	-	-	-	-	-	Healthy
9	50	male	Distal colon	-	-	-	-	-	Healthy
10	38	female	Proximal colon	-	-	-	-	-	Healthy
11	42	female	Distal colon	-	-	-	-	-	Healthy
12	35	female	Distal colon	-	-	-	-	-	Healthy
13	47	male	Proximal colon	-	-	-	-	-	Healthy
14	42	male	Distal colon	-	-	-	-	-	Healthy
15	40	male	Distal colon	-	-	-	-	-	Healthy
16	39	male	Distal colon	-	-	-	-	-	Healthy
17	43	male	Distal colon	-	-	-	-	-	Healthy
18	44	male	Distal colon	-	-	-	-	-	Healthy
19	31	male	Proximal colon	-	-	-	-	-	Healthy
20	34	female	Distal colon	-	-	-	-	-	Healthy
21	39	male	Distal colon	-	-	-	-	-	Healthy
22	42	male	Distal colon	-	-	-	-	-	Healthy
23	40	male	Distal colon	-	-	-	-	-	Healthy

24	47	male	Distal colon	-	-	-	-	-	Healthy
25	44	male	Distal colon	E3	2	12	0.48	Mesalazine + Infliximab	UC
26	53	male	Proximal colon	E3	2	20	1.10	Mesalazine + Vedolizumab	UC
27	39	female	Proximal colon	E3	1	13	0.13	Mesalazine	UC
28	31	male	Distal colon	E3	3	42	2.70	Mesalazine + Vedolizumab	UC
29	49	male	Distal colon	E3	3	45	3.44	Mesalazine + Vedolizumab	UC
30	47	male	Proximal colon	E3	2	10	0.16	Mesalazine + Vedolizumab	UC
31	22	male	Distal colon	E3	3	35	2.09	Mesalazine + Infliximab	UC
32	31	male	Distal colon	E1	2	10	0.44	Mesalazine + Corticosteroids + Infliximab	UC
33	25	male	Distal colon	E1	2	9	0.14	Mesalazine + Vedolizumab	UC
34	44	female	Distal colon	E2	1	18	0.41	Mesalazine + Infliximab	UC
35	36	male	Distal colon	E3	3	13	1.67	Mesalazine + Infliximab	UC
36	23	male	Distal colon	E3	3	7	1.89	Corticosteroids	UC
37	53	male	Distal colon	E3	3	17	0.16	Mesalazine + Vedolizumab	UC
38	42	female	Proximal colon	E3	1	0.27	8.00	Mesalazine + Vedolizumab	UC
39	35	female	Distal colon	E1	2	0.13	18.00	Mesalazine + Vedolizumab	UC
40	42	male	Distal colon	E3	3	32	12.30	Mesalazine + Infliximab	UC
41	47	female	Distal colon	E2	3	45	10.80	Mesalazine + Corticosteroids + Infliximab	UC
42	37	male	Distal colon	E3	3	45	7.39	Mesalazine + Infliximab	UC
43	27	male	Distal colon	E1	2	35	2.37	Mesalazine + Infliximab	UC
44	34	female	Distal colon	E1	0	28	0.12	Mesalazine + Infliximab	UC
45	41	male	Proximal colon	E3	0	25	1.51	Mesalazine + Vedolizumab	UC
46	38	male	Distal colon	E3	2	10	0.36	Mesalazine + Vedolizumab	UC
47	20	male	Distal colon	E1	0	7	<0.2	Mesalazine + Infliximab	UC
48	30	female	Distal colon	E3	2	18	0.21	Mesalazine	UC
49	27	male	Distal colon	E2	2	5	0.58	Mesalazine + Vedolizumab	UC

50	65	Female	Distal colon	E3	3	-	1	Mesalazine	UC
51	63	Female	Distal colon	E3	3	-	101.92	Corticosteroids	UC
52	56	Female	Distal colon	E3	3	-	21.89	Mesalazine + Corticosteroids	UC
53	32	Female	Proximal colon	E3	3	-	119.75	Corticosteroids	UC
54	62	Male	Distal colon	E3	3	-	12.45	Mesalazine	UC
55	52	Female	Distal colon	E2	2	45	50.28	Mesalazine	UC

Table S2. Reagents or oligonucleotides used in this study

REAGENT or RESOURCE	SOURCE	IDENTIFIER
**Antibodies**		
Anti-mouse CD11c-APC	Biolegend	Cat# 117309; RRID: AB_313778
Anti-mouse MHCII-PE	Biolegend	Cat# 107607 RRID: AB_313322
Anti-mouse CD4-APC-Cy7	Biolegend	Cat# 100414; RRID: AB_312699
Anti-mouse CD4-PE-Cy7	eBioscience	Cat# 25-0041-81; RRID: AB_469575
Anti-mouse CD25-PE	Biolegend	Cat# 102008; RRID: AB_312857
Anti-mouse CD11b-PE-Cy7	Invitrogen	Cat# 25-0112-81; RRID: AB_469587
Anti-mouse Ly6G-APC	Biolegend	Cat# 127614; RRID: AB_2227348
Anti-mouse F4/80-FITC	eBioscience	Cat# 11-4801-85; RRID: AB_2637192
Anti-mouse Foxp3-APC	Biolegend	Cat# 320014; RRID: AB_439750
Anti-mouse IL-17A-PE-Cy7	eBioscience	Cat# 25-7177-80; RRID: AB_10717952
Anti-mouse IFN-γ-PerCP/Cy5.5	Biolegend	Cat# 505821; RRID: AB_961361
Anti-mouse IL-4-PE	Biolegend	Cat# 504103;RRID: AB_315317
Anti-Rabbit IgG(H+L)-CoraLite594	Proteintech	Cat# SA00013-4;RRID: AB_2810984
Anti-Rabbit IgG(H+L)-CoraLite488	Proteintech	Cat# SA00013-2;RRID: AB_2797132
Anti-Goat IgG (H+L)-R-PE	Proteintech	Cat# SA00008-3;RRID: AB_2890955
MUC2 antibody	Proteintech	Cat# 27675-1-AP;RRID: AB_2880943
Ki67 antibody	Abcam	Cat# ab264429;RRID: AB_3674140
Cleaved Caspase-3 antibody	Cell Signaling Technology	Cat# 9664;RRID: AB_2070042
Chromogranin A antibody	Abcam	Cat# ab254322;RRID: AB_2910555
SLC36A1 antibody	Proteintech	Cat# 24775-1-AP;RRID: AB_2918086
Gli1 antibody	Proteintech	Cat# 66905-1-Ig;RRID: AB_2882232
tdTomato antibody	Biorbyt	Cat# orb182397;RRID: AB_2687917
**Chemicals, Peptides, and Recombinant Proteins**		
Ampicillin	Sangon Biotech	Cat# A610028-0025
Metronidazole	Sangon Biotech	Cat# A600633-0025
Neomycin	Sangon Biotech	Cat# A610366-0025
Vancomycin	Sangon Biotech	Cat# A600983-0001
Benzydamine	Aladdin	Cat# B129528
Nalidixic acid	Aladdin	Cat# N104920
Forskolin	Selleck	Cat# S2449
SQ22536	Selleck	Cat# S8283
Cyclopamine	Selleck	Cat# S1146
AS1842856	Selleck	Cat# S8222
IWP-2	Selleck	Cat# S7085
DSS	MP Biomedicals	Cat# 0216011090
PBS	Solarbio	Cat# P1020
DMSO	Solarbio	Cat# D8372
DTT	Solarbio	Cat# D1070
Collagenase IV	Sigma	Cat# C5138
Percoll	GE	Cat# 17-0891-01
EDTA	Solarbio	Cat# E1170

OCT	Scigen	Cat# 4586
DAPI	Solarbio	Cat# C0060
4-Guanidinobutyric acid	Sigma	Cat# G6503
**Critical Commercial Assays**		
Foxp3/transcription factor staining buffer sets	eBioscience	Cat# 00-5523-00
Cell activation cocktail with Brefeldin A	Biolegend	Cat# 423303
Zombie NIRTM Fixable Viability Kit	Biolegend	Cat# 423105
Fixation buffer	Biolegend	Cat# 420801
AB-PAS Stain Kit	Solarbio	Cat# G1285
Intracellular staining permeabilization wash buffer	Biolegend	Cat# 421002
Rabbit Two-Step Kit (Rabbit Polymer Detection System)	ZSGB-BIO	Cat# PV-6001
DAB Chromogenic Kit	ZSGB-BIO	Cat# ZLI-9017
Total RNA Extraction Kit	Solarbio	Cat# R1200
RevertAid First Strand cDNA Synthesis Kit	Thermofisher	Cat# K1622
ChamQ Blue Universal SYBR qPCR Master Mix	Vazyme	Cat# Q312
**Experimental Models: Organisms/Strains**		
C57BL/6J wild-type (WT) mice	Academy of Military Medical Science (China)	N/A
Lgr5-CreERT2 mice	GemPharmatech Co., Ltd.(China)	N/A
H11-CAG-LSL-tdTomato mice	GemPharmatech Co., Ltd.(China)	N/A
Muc2$^{\Delta IEC}$ mice	GemPharmatech Co., Ltd.(China)	N/A
**Bacterial Strains and plasmid**		
Prevotella copri	This paper	N/A
Bacteroides fragilis	This paper	N/A
Bacteroides intestinalis	This paper	N/A
Bacteroides stercorirosoris	This paper	N/A
Bacteroides stercoris	This paper	N/A
Bacteroides thetaiotaomicron	This paper	N/A
Bacteroides xylanisolvens	This paper	N/A
Bifidobacterium pseudocatenulatum	This paper	N/A
Bifidobacterium bifidum	This paper	N/A
Bifidobacterium pseudolongum subsp. globosum	This paper	N/A
Coprobacillus cateniformis	This paper	N/A
Beduini massiliensis	This paper	N/A
Clostridium ramosum	This paper	N/A

Flavonifractor plautii	This paper	N/A
Longicatena caecimuris	This paper	N/A
Murimonas intestini	This paper	N/A
Parabacteroides distasonis	This paper	N/A
RIAY_s	This paper	N/A
Akkermansia muciniphila TMU	Lab stored	N/A
Citrobacter rodentium DBS100	ATCC	ATCC 51459
psPAX2	Lab stored	N/A
pMD2.G	Lab stored	N/A
pITA	Lab stored	N/A
pITA-3×FLAG-SLC36A1	This paper	N/A
pLKO.1	Lab stored	N/A
pLKO.1-Scr	This paper	N/A
pLKO.1-sh*SLC36A1*#1	This paper	N/A
pLKO.1-sh*SLC36A1*#2	This paper	N/A
**Oligonucleotides**		
Mouse-*Il1β* F: GGGCTGGACTGTTTCTAATGC	Sangon Biotech	Custom made
Mouse-*Il1β* R: CTTGTGACCCTGAGCGACC	Sangon Biotech	Custom made
Mouse-*TNFα* F: GATCGGTCCCCAAAGGGATG	Sangon Biotech	Custom made
Mouse-*TNFα* R: TTTGCTACGACGTGGGCTAC	Sangon Biotech	Custom made
Mouse-*iNOS* F: GTTCTCAGCCCAACAATACAAGA	Sangon Biotech	Custom made
Mouse-*iNOS* R: GTGGACGGGTCGATGTCAC	Sangon Biotech	Custom made
Mouse-*Cxcl1* F: TGGCTGGGATTCACCTCAAG	Sangon Biotech	Custom made
Mouse-*Cxcl1* R: CCGTTACTTGGGGACACCTT	Sangon Biotech	Custom made
Mouse-*β-actin* F: CACTGTCGAGTCGCGTCCA	Sangon Biotech	Custom made
Mouse-*β-actin* R: GACCCATTCCCACCATCACA	Sangon Biotech	Custom made
Mouse-*Ccl2* F: CACCAGCCAACTCTCACTGAA	Sangon Biotech	Custom made
Mouse-*Ccl2* R: CATTCCTTCTTGGGGTCAGC	Sangon Biotech	Custom made
Mouse-*Muc2* F: AGGGCTCGGAACTCCAGAAA	Sangon Biotech	Custom made
Mouse-*Muc2* R: CCAGGGAATCGGTAGACATCG	Sangon Biotech	Custom made

dnaA F: CAGCACGTGAAGGTGGGGAC	Sangon Biotech	Custom made
dnaA R: CCTTGCGGTTGGCTTCAGAT	Sangon Biotech	Custom made
Mouse-*Lgr5* F: CCTACTCGAAGACTTACCCAGT	Sangon Biotech	Custom made
Mouse-*Lgr5* R: GCATTGGGGTGAATGATAGCA	Sangon Biotech	Custom made
Mouse-*Ascl2* F: AAGCACACCTTGACTGGTACG	Sangon Biotech	Custom made
Mouse-*Ascl2* R: AAGTGGACGTTTGCACCTTCA	Sangon Biotech	Custom made
Mouse-*Sox9* F: GAGCCGGATCTGAAGAGGGA	Sangon Biotech	Custom made
Mouse-*Sox9* R: GCTTGACGTGTGGCTTGTTC	Sangon Biotech	Custom made
Mouse-*Mki67* F: ATCATTGACCGCTCCTTTAGGT	Sangon Biotech	Custom made
Mouse-*Mki67* R: GCTCGCCTTGATGGTTCCT	Sangon Biotech	Custom made
Mouse-*Tff3* F: CCTCTGGCTAATGCTGTTGGT	Sangon Biotech	Custom made
Mouse-*Tff3* R: GGTTGTTACACTGCTCCGATG	Sangon Biotech	Custom made
Mouse-*Atoh1* F: GAGTGGGCTGAGGTAAAAGAGT	Sangon Biotech	Custom made
Mouse-*Atoh1* R: GGTCGGTGCTATCCAGGAG	Sangon Biotech	Custom made
Mouse-*Spdef* F: AAGGCAGCATCAGGAGCAATG	Sangon Biotech	Custom made
Mouse-*Spdef* R: CTGTCAATGACGGGACACTG	Sangon Biotech	Custom made
Mouse-*Gli1* F: CCAAGCCAACTTTATGTCAGGG	Sangon Biotech	Custom made
Mouse-*Gli1* R: AGCCCGCTTCTTTGTTAATTTGA	Sangon Biotech	Custom made
Mouse-*Ptch1* F: CTCTTGGTGTTGGTGTGGATG	Sangon Biotech	Custom made
Mouse-*Ptch1* R: AGGCGGTGACATTGCTGAT	Sangon Biotech	Custom made
shScr F: CCGGCAACAAGATGAAGAGCACCAACTCGAGTTGGTGCTCTTCATCTTGTTGTTTTTG	Sangon Biotech	Custom made

shScr R: AATTCAAAAACAACAAGATGAAGAGCACCAACTCGAGTTGGTGCTCTTCATCTTGTTG	Sangon Biotech	Custom made
Sh*SLC36A1*#1 F: CCGGCGGTGATGTATGGACTAGAATCTCGAGATTCTAGTCCATACATCACCGTTTTTG	Sangon Biotech	Custom made
Sh*SLC36A1*#1 R: AATTCAAAAACGGTGATGTATGGACTAGAATCTCGAGATTCTAGTCCATACATCACCG	Sangon Biotech	Custom made
ShSLC36A1#2 F: CCGGCCTGATCATGATATACCAGTTCTCGAGAACTGGTATATCATGATCAGGTTTTTG	Sangon Biotech	Custom made
Sh*SLC36A1*#2 R: AATTCAAAAACCTGATCATGATATACCAGTTCTCGAGAACTGGTATATCATGATCAGG	Sangon Biotech	Custom made
Cloning SLC36A1 into pITA-3×FLAG F: ATGACAAGCTTGAGGTTGGGATGTCCACACAGAGGCTTCG	Sangon Biotech	Custom made
Cloning SLC36A1 into pITA-3×FLAG R: AGGACCTGCCAGAAAGGCTGTTATATGAAGGCACTGGTGGAATTGGTAGAG	Sangon Biotech	Custom made
**Software and Algorithms**		
FlowJo10.9	TreeStar	https://www.flowjo.com/ RRID: SCR_008520
Prism 8	GraphPad software	https://www.graphpad.com/ RRID: SCR_002798
Image Pro Plus	Media Cybernetics	http://www.mediacy.com/imageproplus/ RRID:SCR_007369
**Deposited data**		
16S rRNA gene sequencing	BioProject	PRJNA1298487; PRJNA1298490
Single-cell RNA sequencing	BioProject	PRJNA1298199
Non-targeted metabolomics	Metabolomics Workbench	PR002560
